# Variable Responses to Carbon Utilization between Planktonic and Biofilm Cells of a Human Carrier Strain of *Salmonella enterica* Serovar Typhi

**DOI:** 10.1371/journal.pone.0126207

**Published:** 2015-05-06

**Authors:** Kalaivani Kalai Chelvam, Kien Pong Yap, Lay Ching Chai, Kwai Lin Thong

**Affiliations:** Institute of Biological Sciences, Faculty of Science, University of Malaya, Kuala Lumpur, Malaysia; University of Osnabrueck, GERMANY

## Abstract

*Salmonella enterica* serovar Typhi (*S*. Typhi) is a foodborne pathogen that causes typhoid fever and infects only humans. The ability of *S*. Typhi to survive outside the human host remains unclear, particularly in human carrier strains. In this study, we have investigated the catabolic activity of a human carrier *S*. Typhi strain in both planktonic and biofilm cells using the high-throughput Biolog Phenotype MicroArray, Minimum Biofilm Eradication Concentration (MBEC) biofilm inoculator (96-well peg lid) and whole genome sequence data. Additional strains of *S*. Typhi were tested to further validate the variation of catabolism in selected carbon substrates in the different bacterial growth phases. The analyzes of the carbon utilization data indicated that planktonic cells of the carrier strain, *S*. Typhi CR0044 could utilize a broader range of carbon substrates compared to biofilm cells. Pyruvic acid and succinic acid which are related to energy metabolism were actively catabolised in the planktonic stage compared to biofilm stage. On the other hand, glycerol, L-fucose, L-rhamnose (carbohydrates) and D-threonine (amino acid) were more actively catabolised by biofilm cells compared to planktonic cells. Notably, dextrin and pectin could induce strong biofilm formation in the human carrier strain of *S*. Typhi. However, pectin could not induce formation of biofilm in the other *S*. Typhi strains. Phenome data showed the utilization of certain carbon substrates which was supported by the presence of the catabolism-associated genes in *S*. Typhi CR0044. In conclusion, the findings showed the differential carbon utilization between planktonic and biofilm cells of a *S*. Typhi human carrier strain. The differences found in the carbon utilization profiles suggested that *S*. Typhi uses substrates mainly found in the human biliary mucus glycoprotein, gallbladder, liver and cortex of the kidney of the human host. The observed diversity in the carbon catabolism profiles among different *S*. Typhi strains has suggested the possible involvement of various metabolic pathways that might be related to the virulence and pathogenesis of this host-restricted human pathogen. The data serve as a caveat for future *in-vivo* studies to investigate the carbon metabolic activity to the pathogenesis of *S*. Typhi.

## Introduction

*Salmonella enterica* serovar Typhi (*S*. Typhi) causes typhoid fever. Overall, there are about 16 million cases with 600,000 related deaths worldwide [1]. *S*. Typhi is a rod-shaped, Gram-negative bacterium, which is pathogenic to humans only and does not infect plants or animals [2]. Infection takes place when food or water contaminated with *S*. Typhi is ingested via faecal-oral route. Most patients eventually recover from the illness although some may continue to shed *S*. Typhi in their stools as carriers [2]. There is a great interest in the ability to form biofilms. Biofilm formation ability in bacteria has gained numerous attention globally, as more and more studies have associated biofilm formation ability in bacteria to emergence of many antibiotic-resistance and persistent infections in humans [3–4]. This is especially true in *S*. Typhi, in which its ability to form strong biofilm on gallstones [5] and gallbladder epithelium [6] has been suggested to play an important role in long-term persistence in an asymptomatic carrier state. Asymptomatic typhoid human carrier is believed to be the source of transmission and persistence of typhoid fever in areas of endemicity [6]. Despite the fact that many intensive studies had been conducted to understand the pathogenesis of *S*. Typhi, little is known about the mechanisms of colonization and persistence of the pathogen in a chronic human carrier. Therefore, the main aim of this current study is to investigate if *S*. Typhi would show differentiation in its metabolic preferences in planktonic and biofilm state and also the types of carbons substrates that would induce the transformation from planktonic state into biofilm state. To achieve the objective, we have selected a human carrier *S*. Typhi strain, CR0044 that have the ability to form strong biofilm [7] for this work. CR0044 was isolated from an asymptomatic human carrier from Kelantan, Malaysia that is highly endemic for typhoid fever. We believe that this carrier strain would serve as a good model for this study to explore the adaptive mechanism of *S*. Typhi in two different physiology states, biofilm and planktonic. Furthermore, the ability of *S*. Typhi to survive outside the human host that remains largely unclear, would be investigated. In this study, differential carbon catabolism of the strain in planktonic and biofilm stages was measured using the high-throughput Biolog Phenotype MicroArray (PM) and Minimum Biofilm Eradication Concentration (MBEC) biofilm inoculator (96-well peg lid) [8]. Metabolic activity was measured by detecting a color change in the tetrazolium dye when there is bacterial respiration in each well. The described method can easily be performed and it provides important insights into the metabolic properties of *S*. Typhi in planktonic and biofilm cells, as well as carbon substrates that induce the biofilm formation. PM technology has been reported to be used in many studies to reveal metabolic properties of various bacteria [9–13]. However, studies describing the metabolic activity of bacterial biofilms are limited [14]. The objectives of this work were to (i) determine the differences in carbon catabolism of a human carrier *S*. Typhi strain during planktonic and biofilm state; and (ii) identify the specific carbon substrates that will induce the transition of planktonic cells to form biofilm.

## Materials and Methods

### Bacterial strains

*S*. Typhi CR0044 strain was isolated from the stool sample of a food handler in Kelantan, Malaysia, in 2007 [15]. Previously, we reported that CR0044 is motile and has the ability to form robust biofilms [7]. We used additional strains of *S*. Typhi, namely BL191, BL196, S5680, ST33, ST280, STVC1681 and STVC3121 (S1 Table) to further validate the variation of catabolism in selected carbon substrates during the different growth phases. Except for STVC1681, which was isolated from contaminated sewage water [16], all the other strains used for validation were isolated from typhoid patients. The strains were retrieved from—80°C stock culture, and reconfirmed as *S*. Typhi using an in-house PCR assay. We use the direct PCR targeting the *hil*A gene of *Salmonella* and a flagellin gene for *Salmonella* Typhi to confirm the purity of the strains [17].

### Phenotype MicroArray testing

The Phenotype MicroArray (PM) assay was performed using the 96-well PM1 and PM2A plates from Biolog Inc. (Hayward, CA) [18] to test the catabolic ability of the *S*. Typhi human carrier strain to 190 carbon sources. The principle of the test relies on reduction of the redox dye tetrazolium violet by metabolically-active bacterial cells. PM1 and PM2A microplates comprise of 190 carbon compounds of alcohol, amide, amine, amino acid, carbohydrate, carboxylic acid, ester, fatty acid and polymer (S2 Table). The carbon substrate, dye, and nutrients were supplied in each well in a dried-film form which was reconstituted upon addition of an aliquot of bacterial cultures (100 μl) [19].

### Detection of the metabolic activity in planktonic stage

Cultures of strain CR0044 were plated onto solid lysogeny broth medium (LB) and incubated at 37°C for 18 h. Then, five to ten single colonies were picked up with a moistened cotton swab and then resuspended in inoculating fluid IF-0a (Biolog) to a cell density of 85% transmittance. Equal volume of 1% dye A (Biolog) (vol/vol) was added to the cell suspension. One hundred microlitres of the mixture were loaded into each well on PM microplate PM1 and PM2A. The plates were then incubated at 37°C in an OmniLog reader. The readings were recorded at 48 h and the data were analyzed using OmniLog PM software. The PM data analyzes were performed with the area under the growth curve (AUC) [20]. During data processing, the option of A1 zero (negative control) was selected to subtract the background noise in each of the 96-well plates. Plates were analyzed in duplicates.

### Biofilm inoculator assay

MBEC is a high-throughput screening assay used to check the viability of biofilm produced on the 96-well peg lid (MBEC Biofilms Technology Ltd., Calgary Alberta, Canada) [21]. The MBEC assay was carried out according to the methods and procedures outlined by the manufacturer (Product: P & G Panel; Lot No: 13030018 and 13030029).

### Detection of the metabolic activity in biofilm stage

*S*. Typhi CR0044 was streaked on LB agar and incubated at 37°C for 24 h. Two to three *S*. Typhi fresh colonies were transferred to a flask containing 100 ml LB broth and grown overnight until it reached the late exponential phase at Optical Density of 0.1 (OD_590nm_ = 0.1). The adjusted bacterial suspension was further diluted 10x to achieve a cell density of approximately 10^6^ CFU ml^-1^ prior inoculation into MBEC Biofilm Inoculator. *S*. Typhi biofilm was allowed to grow on each peg for 24 h at 37°C. The peg lid was then transferred into PM microplate PM1 and PM2A. PM microplates were incubated at 37°C in an OmniLog reader. The readings were recorded and analyzed using OmniLog PM software.

### Detection of the carbon sources that induce biofilm formation

*S*. Typhi CR0044 was cultured in LB media and incubated at 37°C for 24 h. Five to ten single colonies were picked up with a cotton swab moistened with inoculating fluid IF-0 (Biolog) and then resuspended into glass tubes to reach a cell density of 85% transmittance. One hundred microlitres of the bacterial suspension were loaded into each well on PM microplate PM1 and PM2A. At every 6 h interval, one set of PM microplates (PM1 and PM2A) was removed from incubator for biofilm quantification. The content of each well was removed and the non-adhered cells in each well were removed by vigorous tapping of the inverted microtiter plate on absorbent paper [22]. Subsequently, adhered biofilm cells were heat-fixed in an oven for 30 min at 80°C. Quantification of biofilm formation was carried out by staining of adhered cells with 220 μl of crystal violet stain (0.5%) for 1 min, followed by destaining solution (ethanol/acetone, 80:20%) for 15 min. The absorbance was measured at OD_590nm_ wavelength [22].

### Validation of Phenotype MicroArray data

After completion of PM analysis, six carbon substrates (glycogen, laminarin, mannan, chondroitin sulfate C, pectin and dextrin) were randomly selected for further validation of the metabolic activity measured with Biolog PM technology. To determine the carbon utilization by planktonic *S*. Typhi cells, five to six single colonies of *S*. Typhi CR0044 were inoculated into 5 ml of IF-0a inoculating fluid (Biolog) supplemented with 10 mM of the selected carbon substrate and incubated at 37°C for 24 h in a shaking water bath (Memmert, Germany). A positive result is determined by the observation of cellular turbidity in the particular carbon substrates. For detection of metabolic activity in biofilm stage and carbon substrates that induce biofilm formation, similar procedures as described earlier was used, except that the selected carbon substrates (10 mM for biofilm stage; 20 mM for biofilm formation) were added manually into sterile empty 96-well microtiter plates [23]. Then, carbon substrates that were found to be utilized differently by CR0044 when grown as planktonic cells, biofilms cells and inducing the formation of biofilm, were further tested using methods described earlier with additional seven strains of *S*. Typhi. The carbon substrates selected for further confirmation were: D-threonine, D-melibiose, glycerol, L-rhamnose, L-lactic acid, succinic acid and pectin. All assays were performed in duplicate to ensure reproducibility of results.

### Genome mining and metabolic pathway modeling

The genome of human carrier strain of *S*. Typhi CR0044 was assembled and annotated as previously described (GenBank Accession number: AKZO00000000.1) [15]. Annotated protein sequences were mapped against KEGG databases and used for KEGG orthology and metabolic pathway assignments [24]. The respective ortholog tables, KEGG module and KEGG compound were extracted to construct metabolic maps (S1 File).

### Data analysis

The Biolog OmniLog PM software was used to export the data for each run. A final Area Under the Curve (AUC) value for all the runs was calculated manually using Microsoft Excel. At the same time, OD or absorbance readings within each well were taken at 0 h, 6 h, 12 h, 24 h, 36 h and 48 h. Essentially, the dye reduction and OD values were used to calculate mean values and standard deviation (SD) throughout the 48 h. Microsoft Excel was used to construct the Scatter plot to check for normalization of data. All assays were performed in duplicates. IBM SPSS Statistics version 22.0.0 was used to perform basic statistical parameters.

## Results and Discussion

### Limited carbon catabolism activity in *S*. Typhi CR0044 during biofilm growth stage

**Fig 1:**
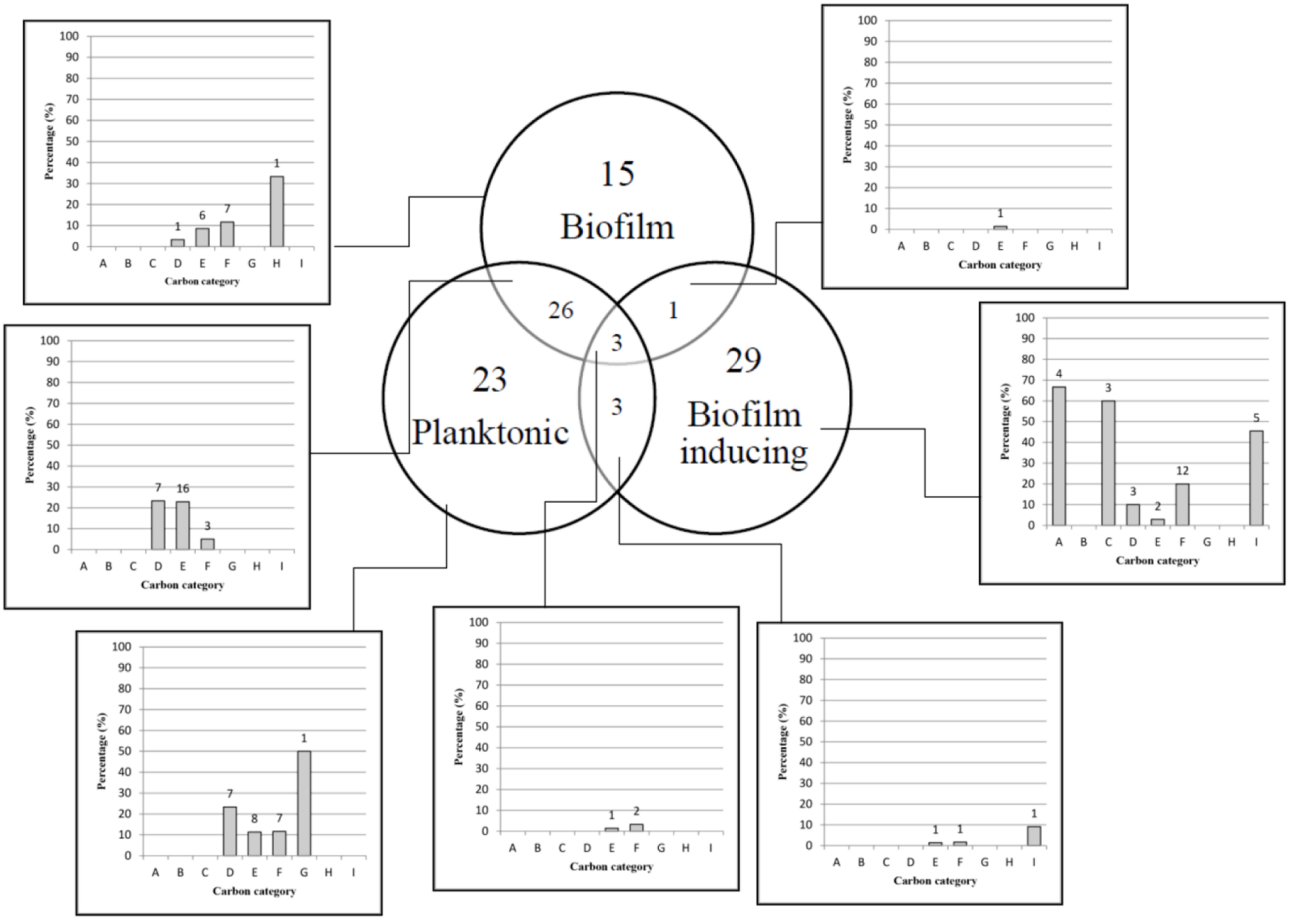
Venn diagram showing carbon substrates catabolised by *S*. Typhi human carrier strain CR0044 in 3 different bacterial growth stages; planktonic, biofilm and inducing transition of planktonic cells to form biofilm. A total of 190 carbon substrates were tested. A: Alcohol, B: Amide, C: Amine, D: Amino acid, E: Carbohydrate, F: Carboxylic acid, G: Ester, H: Fatty acid, I: Polymer. Y-axis indicates the percentage of carbon utilized in planktonic, biofilm and biofilm inducing *S*. Typhi bacterial growth stages. X-axis shows the carbon category for each carbon substrate tested. The Venn diagram was obtained based on the Average Growth Curve (AUC) area and was classified into a combination of 6 different bacterial growth stages: growth only in planktonic; only in biofilm; inducing biofilm formation; planktonic and biofilm only; planktonic and inducing biofilm formation only; biofilm and inducing biofilm formation only; all 3 stages of bacterial growth planktonic, biofilm and inducing biofilm formation. *S*. Typhi biofilm growth stage was tested using the 96-well peg lid on Phenotype MicroArray plate for 48 h. The biofilm inducing experiment was conducted using 0.5% crystal violet stain and absorbance was measured at wavelength OD _590nm_ every 6 h.

**Fig 2:**
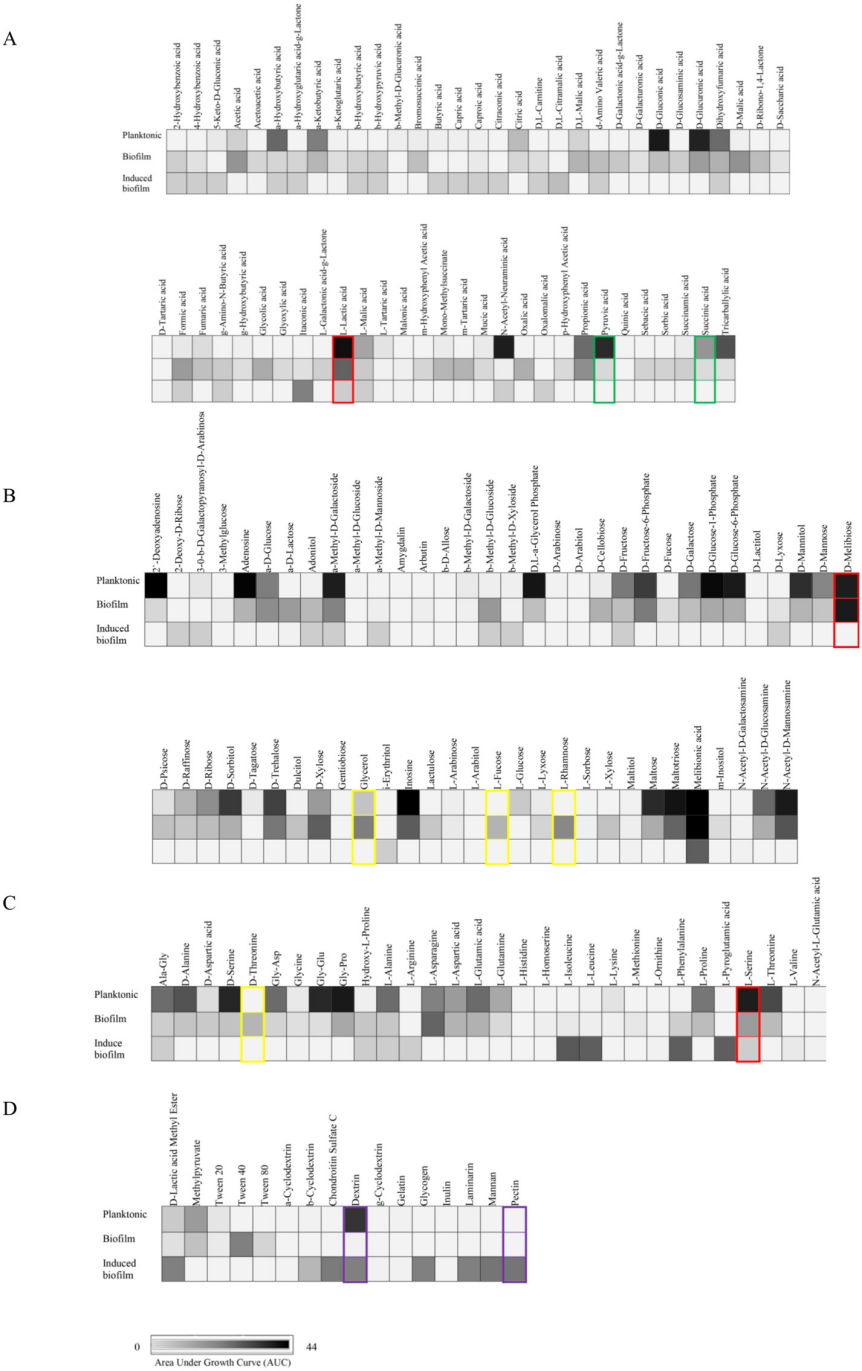
Carbon substrates utilized by *S*. Typhi human carrier strain, CR0044. Fig 2A. Carboxylic acid; Fig 2B. Carbohydrate; Fig 2C. Amino acid; Fig 2D. Ester, fatty acid and polymer. Area under the growth curve values of substrates utilized by *S*. Typhi strain CR0044 was determined using Biolog Phenotype MicroArray plates PM1 and PM2. The maximal kinetic curve height was expressed as a grayscale ranging from 0 (light gray) to 44 (black) area under the curve units. Color highlights show differences between biofilm and planktonic *S*. Typhi; green (planktonic only), yellow (biofilm only), red (both planktonic and biofilm), purple (induced biofilm). Phenotypes < 0 were considered negative.

Overall, CR0044 in biofilm stage was metabolically less active than in planktonic stage. Out of 190 carbon substrates tested, only 15 substrates (7.9%) were utilized during biofilm growth stage compared to 23 substrates (12%) in planktonic growth stage (Fig 1). Interestingly, pyruvic acid and succinic acid that are associated with energy metabolism were more actively catabolised in the planktonic stage compared to biofilm stage (Fig 2A). In this study, L-lactic acid, D-melibiose and L-serine were utilized by CR0044 in both planktonic and biofilm stages, but did not induce the formation of biofilm (Fig 2B and 2C). Sequence analysis of the genome of CR0044 showed the presence of catabolic genes for pyruvic acid, succinic acid, L-serine, and L-lactic acid (S3 Table). Pyruvic acid, L-serine and L-lactic acid are also the end products of gluconeogenesis and glycolysis [25]. The accessibility of pyruvic acid, L-serine and L-lactic acid substrates in the cortex of human kidney suggests possible transient colonization of *S*. Typhi, in this organ, which causes dissemination of the pathogen via urine [26]. Biofilm has a lower carbon catabolism rate because sessile bacteria in this development stage have nutrient starvation and thus develop very slowly and have less motility [27] but planktonic cells are highly motile, have easy access to nutrients and multiply quickly [28–29]. In a separate independent assay, L-lactic acid was found to be utilized by both planktonic cells and biofilm cells of all the seven clinical *S*. Typhi strains. Succinic acid was catabolized by four out of seven strains when they were grown as planktonic cells, but none showed utilization when they were in biofilm stage (S4 Table). L-serine, L-fucose and pyruvic acid were not further tested in this study due to unavailability of these carbon substrates in the laboratory. In the future, metabolomic analysis may clarify the effect of nutrients during infection under the planktonic and biofilm growth conditions.

### Glycerol, L-fucose, L-rhamnose and D-threonine were catabolised only by biofilm but not planktonic cells of *S*. Typhi

CR0044 strain was able to uptake and catabolise glycerol, L-fucose, L-rhamnose (carbohydrates) and D-threonine (amino acid) only in biofilm stage (Fig 2B and 2C). This was supported by the presence of catabolic genes for glycerol, L-fucose, L-rhamnose and D-threonine in the CR0044 genome (S3 Table). The ability to utilize these carbon substrates by biofilm but not planktonic cells of *S*. Typhi could indicate that the corresponding catabolic genes were only activated to allow bacteria to colonize the liver. The human biliary mucus glycoprotein is composed of carbohydrates (79%) and amino acids (21%) in which threonine, serine, and proline constituted 43% by weight of the protein content [30], while the major sugar moieties fucose, galactose, N-acetylglucosamine and N-acetylgalactosamine in mucus glycoproteins, made up 95% of the carbohydrates [30–31]. Kaiser et al. [32] demonstrated that fucose metabolism is likely to occur in *S*. Typhimurium infection *in vivo*. Glycerol is found in the liver and kidneys of the human body [25]. During infection, variable factors such as microbiota composition, nutrient supplement availability and bacterial population may influence the metabolic activity [32]. However, when the differential utilization of these four carbon substrates were tested on more *S*. Typhi strains, only glycerol was found to be able to support growth of *S*. Typhi in biofilm stage but not observed for L-rhamnose and L-threonine (L-fucose was not tested). Furthermore, glycerol was shown to be catabolized by these seven clinical strains of *S*. Typhi in planktonic stage but not in CR0044. Whether this differential utilization is due to the fact that CR0044 is a human carrier strain and the other strains were isolated from the blood or stool samples of typhoid patients, further study is warranted. Nonetheless, the metabolic differences between planktonic and biofilm states observed in this study suggested that the pathogen apply different adaptation mechanisms to colonize and persist in various niches in the host body during the course of infection. Further studies are required to analyze the factors that influence *S*. Typhi metabolism and how the course of infection may be affected.

### Dextrin and pectin induced the transition of CR0044 from planktonic to biofilm stage

Several carbon substrates such as simple sugars and sugar derivatives induced the formation of biofilm in *S*. Typhi CR0044 human carrier strain. The results showed that 45.5% (5 out of 11) polymers could induce strong biofilm formation in CR0044 but were not catabolised in planktonic and biofilm stage. Interestingly, dextrin and pectin induced the biofilm formation but only dextrin supported the growth of planktonic cells (Fig 2D). Pectin is a plant polysaccharide consisting of α-D-galacturonic acid and is a component of the primary cell walls of terrestrial plants [33]. Dextrins are produced in the human body as a result of enzymatic digestion of starch [34]. The KEGG pathway in the Supplementary S1 File showed that the enzymes produced for dextrin and pectin utilization were present in the CR0044 genome under the starch and sucrose metabolism (S1 File). The genes and enzymes were sty:STY4134 *malS*; alpha-amylase (EC:3.2.1.1) and sty:STY0819 *ybhC*; pectinesterase (EC:3.1.1.11). Similar KEGG pathway patterns were observed in the other complete genomes of *S*. Typhi strains CT18 [35], Ty2 [36] and P-stx-12 [37]. The *ybhC* and *malS* genes sequences were BLASTed against the NCBI non-redundant database and the results showed both the genes have 100% identity to all *S*. Typhi reference genomes, CT18, Ty2 and P-stx-12 (BLASTP, Query coverage 100%, E-value 0.0). However, pectin was not able to induce formation of biofilm in the other seven *S*. Typhi strains tested using a separate assay (S4 Table; dextrin was not tested in this study). We could not ascertain if the inability to induce the formation of biofilm with exposure to pectin in the other strains is due to strain variation or if the ability to form biofilm in exposure to pectin and dextrin granted the strain special advantage to survive in the environment. Further studies are required to examine whether the capability of *S*. Typhi to utilize dextrin and pectin contributes to the transient survival mechanism in the environment during human to human transmission.

## Conclusions

In conclusion, the study showed variations in the carbon substrates utilized by the planktonic and biofilm cells of the *S*. Typhi human carrier strain, CR0044. The differences found in the carbon utilization profiles suggested that this human carrier strain of *S*. Typhi used substrates found mainly in biliary mucus glycoprotein, gallbladder, liver and cortex of the kidney of the human host. Certain carbon substrates were activated during biofilm growth stage only. There is a high possibility that these substrates promote persistence by forming biofilm in the gallbladder or liver. Pectin substrate produced strong biofilm formation in *S*. Typhi human carrier strain only, which suggests the transient survival of this strain in the environment before transmission to humans. The diversity observed in the carbon catabolism profiles among different *S*. Typhi strains has suggested the possible involvement of various metabolic pathways that might be related to the virulence and pathogenesis of this host-restricted human pathogen. The phenome data for differential utilization of carbon substrates was supported by the genome data, catabolism-associated genes in human carrier *S*. Typhi CR0044. The data serve as a caveat for future *in vivo* studies to explore the carbon metabolic activity associated to pathogenesis of *S*. Typhi.

## Limitations of the study

The limitations found in this study are the nature of the Biolog plate contents which makes the exact determinations of nutrients difficult. Moreover, in some cases the concentrations of nutrients may be excessively low or in a manner unavailable to the organism tested. In particular, environmental conditions such as temperature, pH, salinity and atmosphere are modified. In addition, the availability of human carrier strains is limited as the isolation and collection of *S*. Typhi strains from asymptomatic typhoid carriers require collection and culture of multiple faecal samples over the period of at least a year.

## Supporting Information

S1 File KEGG metabolic pathway maps constructed from the genome of the *S*. Typhi human carrier strain CR0044. (ZIP)

S1 Table *Salmonella* enterica serovar Typhi strains used in this study. (PDF)

S2 Table A list of all the conditions/ substrates in PM wells for plates PM1 and PM2A. One-hundred and ninety carbon sources are used in the profiling of the *S*. Typhi human carrier strain, CR0044. (PDF)

S3 Table Raw Phenotype MicroArray data and presence/ absence of catabolism- related genes in the *S*. Typhi human carrier strain CR0044. (PDF)

S4 Table Selected carbon substrates utilized by *Salmonella* Typhi strains in 3 different bacterial growth stages; planktonic, biofilm and inducing transition of planktonic cells to form biofilm. (PDF)
