# Supplementary figures and images for: Variable Responses to Carbon Utilization between Planktonic and Biofilm Cells of a Human Carrier Strain of Salmonella enterica Serovar Typhi
Source: PLoS One. 2015 May 6;10(5):e0126207. doi: 10.1371/journal.pone.0126207 (PMC4422432; doi:10.1371/journal.pone.0126207)

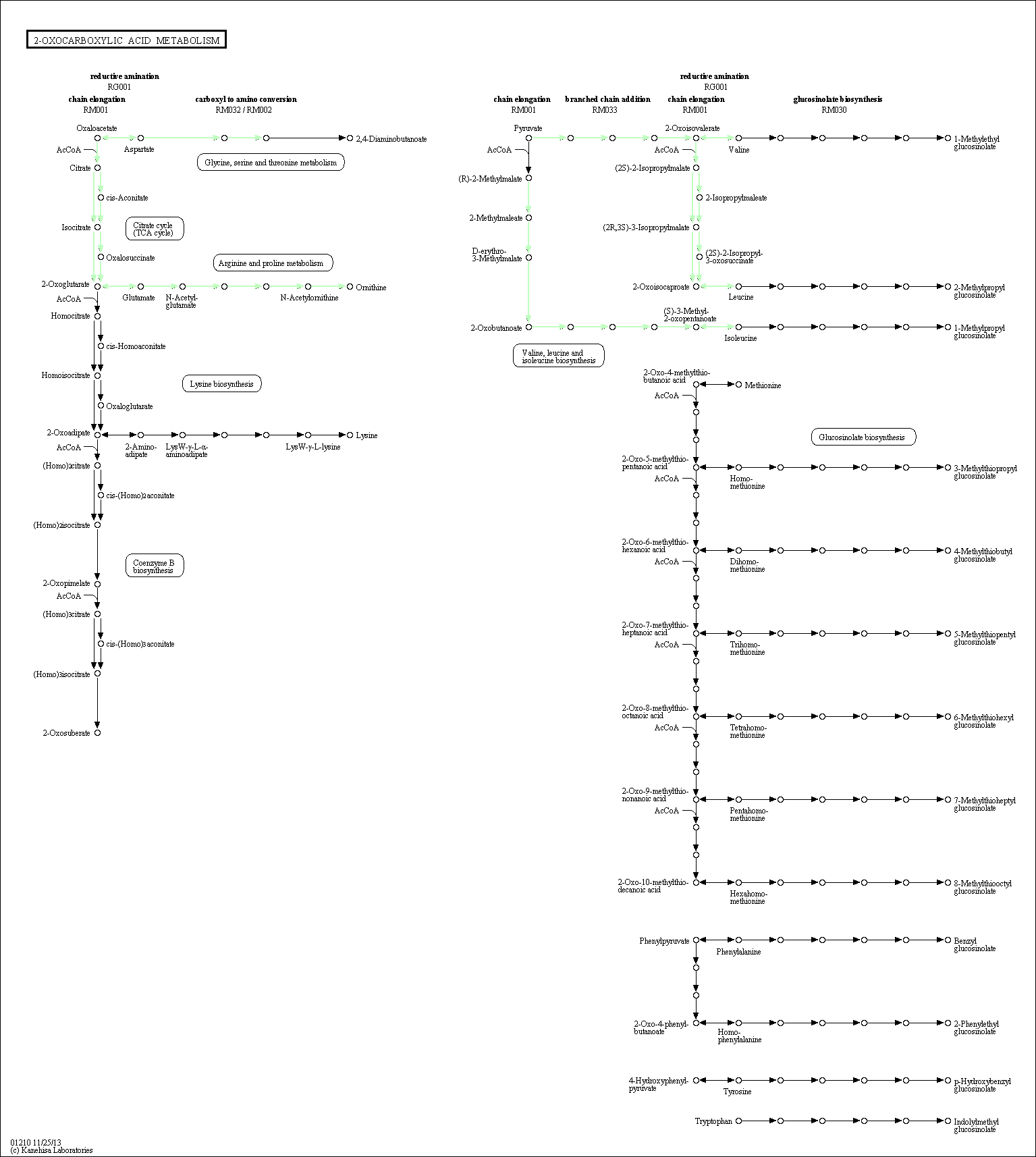

Supplement: S1 File — (ZIP) [file pone.0126207.s001.zip › S1 File/2-Oxocarboxylic Acid Metabolism.png]

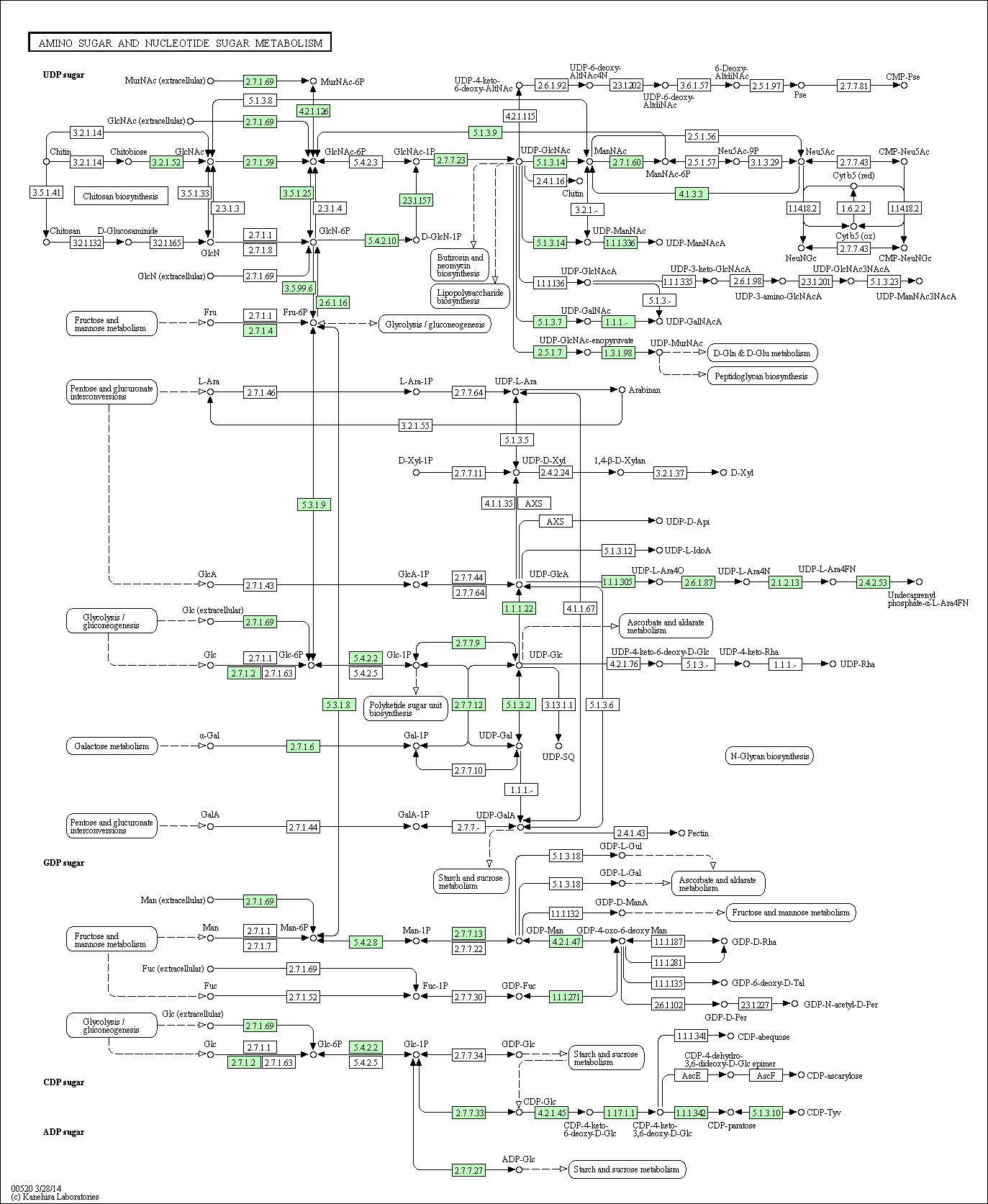

Supplement: S1 File — (ZIP) [file pone.0126207.s001.zip › S1 File/Amino Sugar and Nucleotide Sugar Metabolism.png]

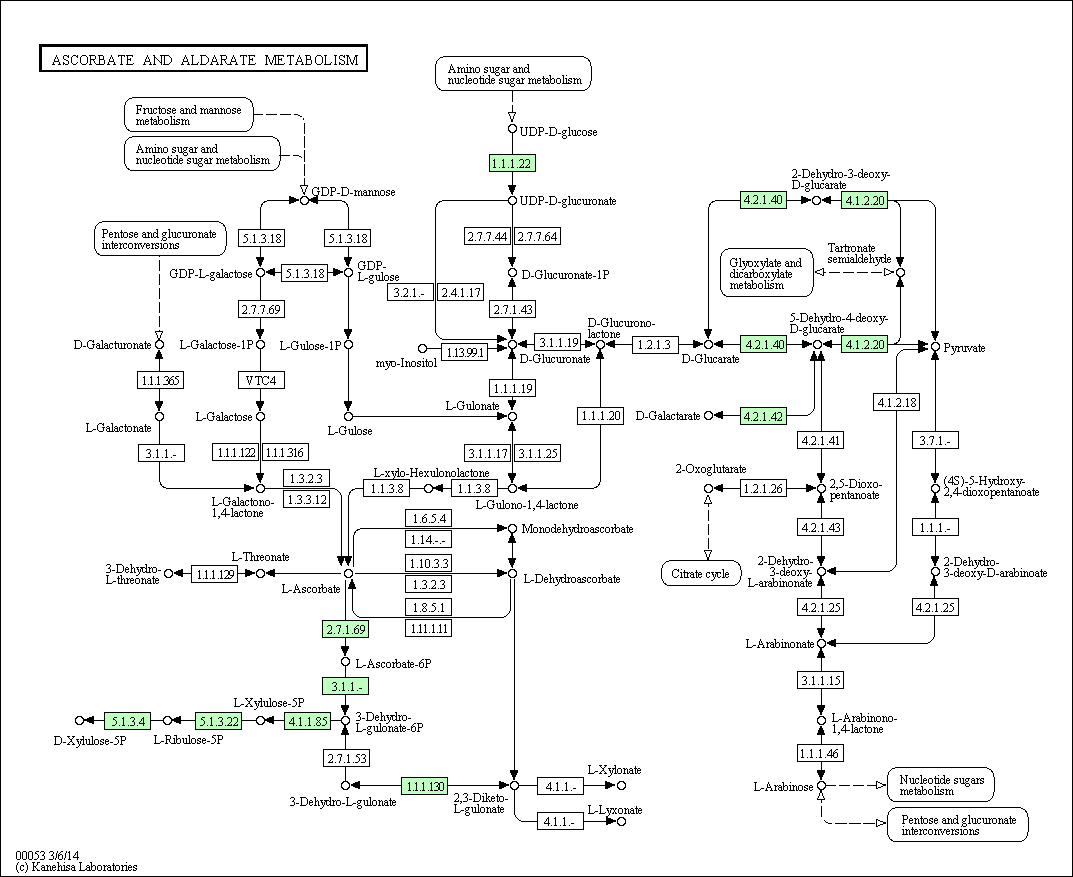

Supplement: S1 File — (ZIP) [file pone.0126207.s001.zip › S1 File/Ascorbate and Aldarate Metabolism.png]

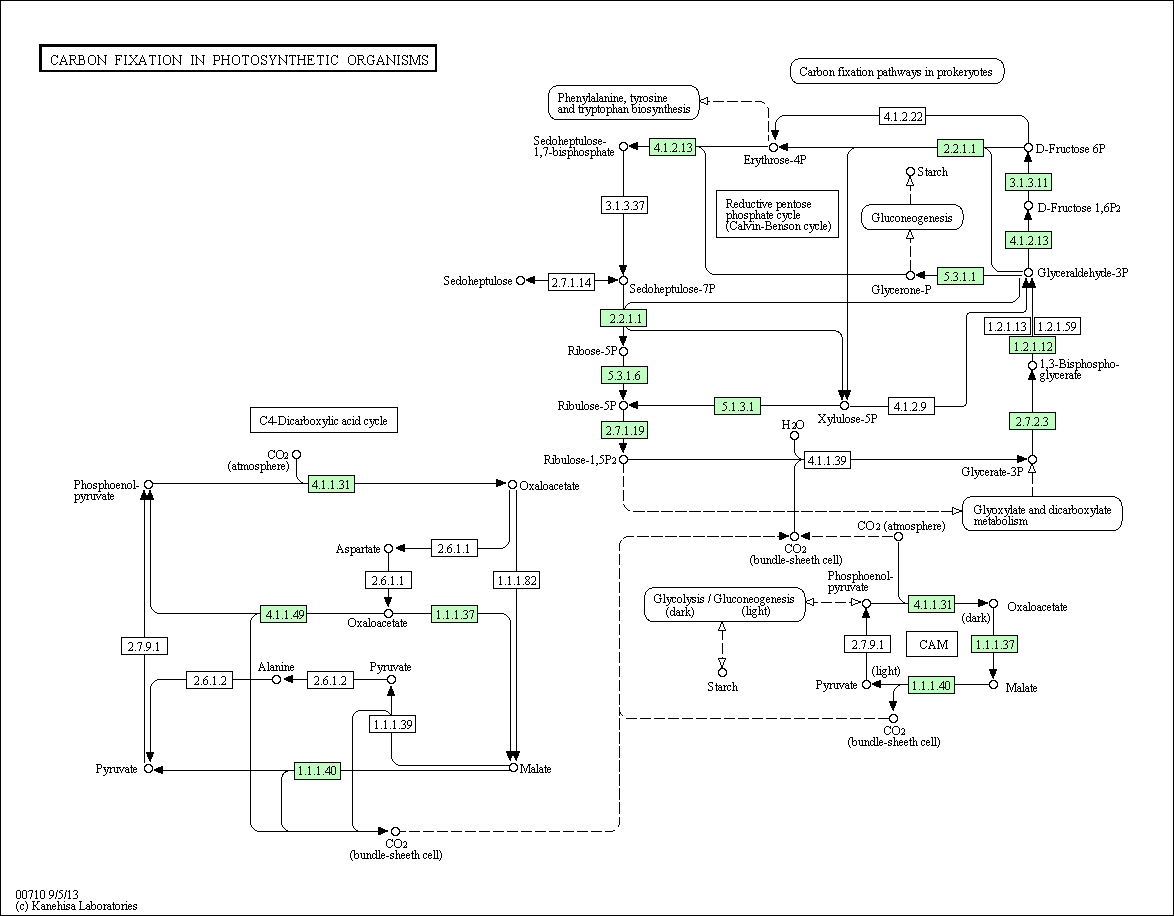

Supplement: S1 File — (ZIP) [file pone.0126207.s001.zip › S1 File/Carbon Fixation in Photosynthetic Organisms.png]

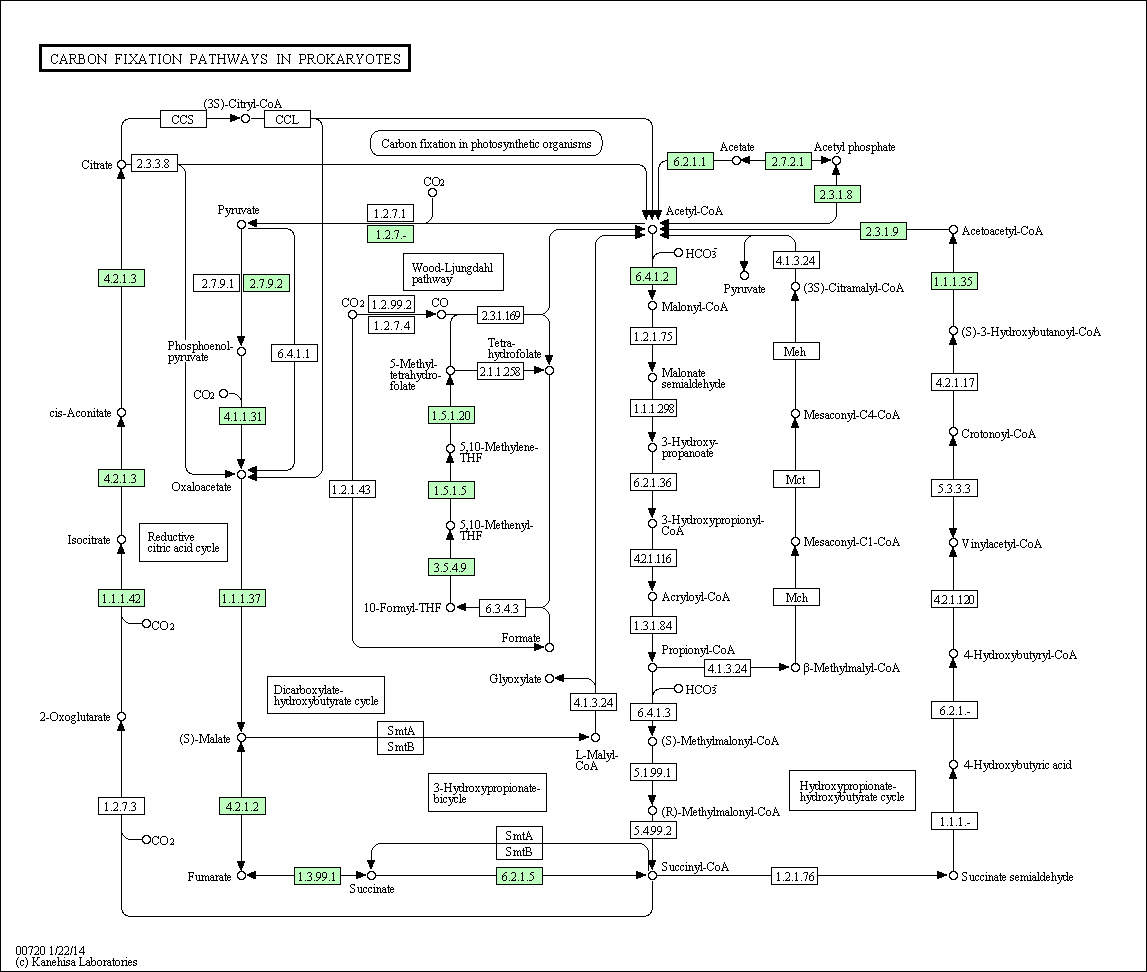

Supplement: S1 File — (ZIP) [file pone.0126207.s001.zip › S1 File/Carbon Fixation Pathways in Prokaryotes.png]

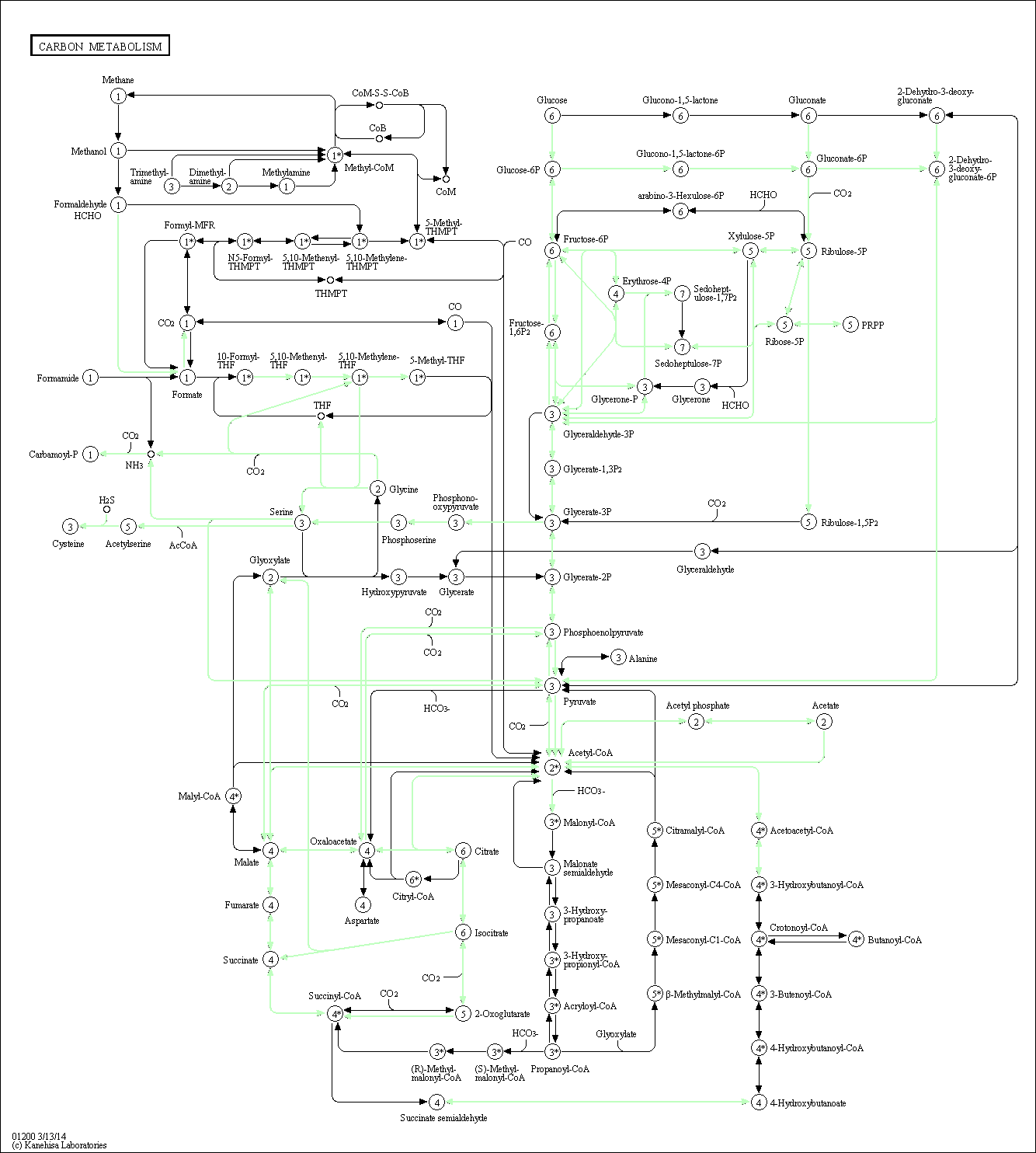

Supplement: S1 File — (ZIP) [file pone.0126207.s001.zip › S1 File/Carbon Metabolism.png]

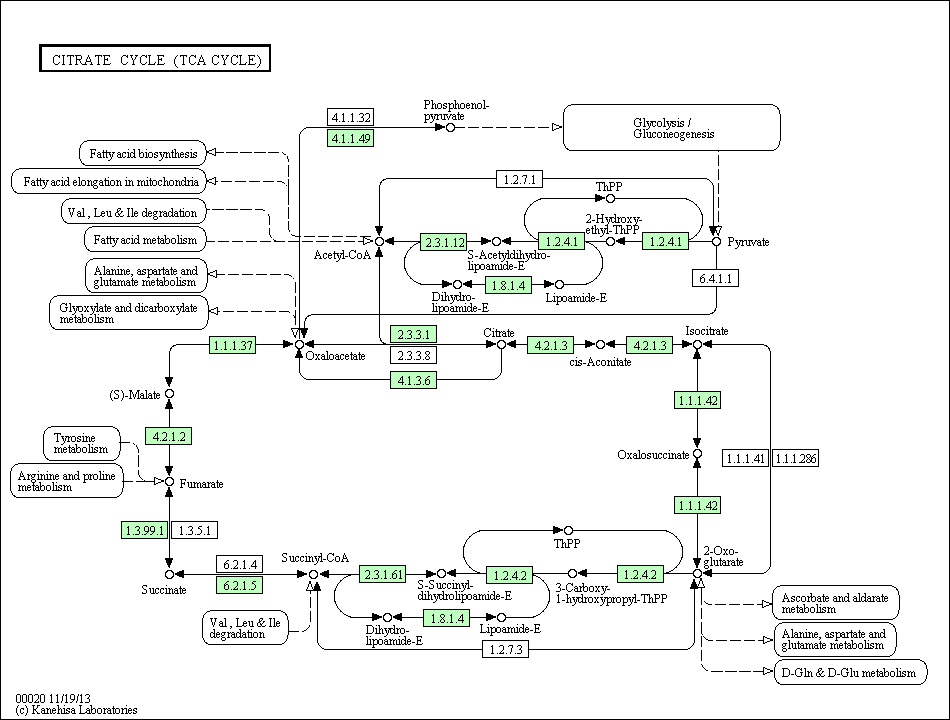

Supplement: S1 File — (ZIP) [file pone.0126207.s001.zip › S1 File/Citrate Cycle (TCA cycle).png]

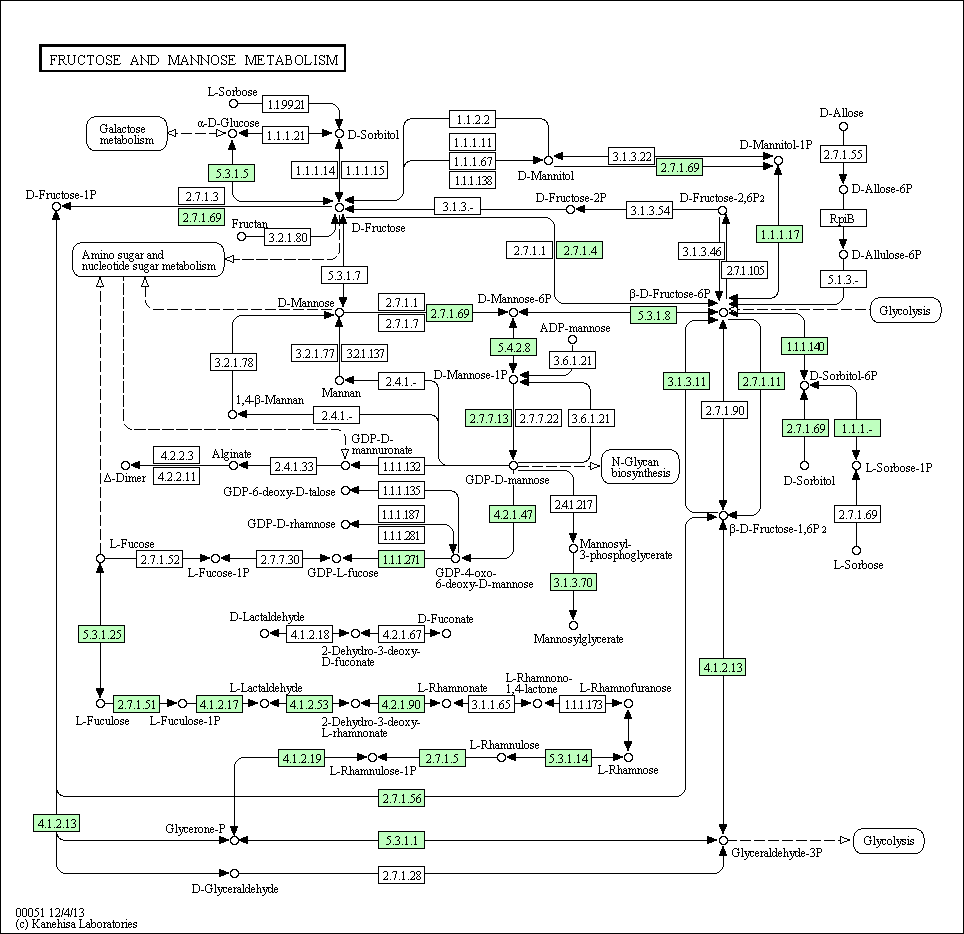

Supplement: S1 File — (ZIP) [file pone.0126207.s001.zip › S1 File/Fructose and Mannose Metabolism.png]

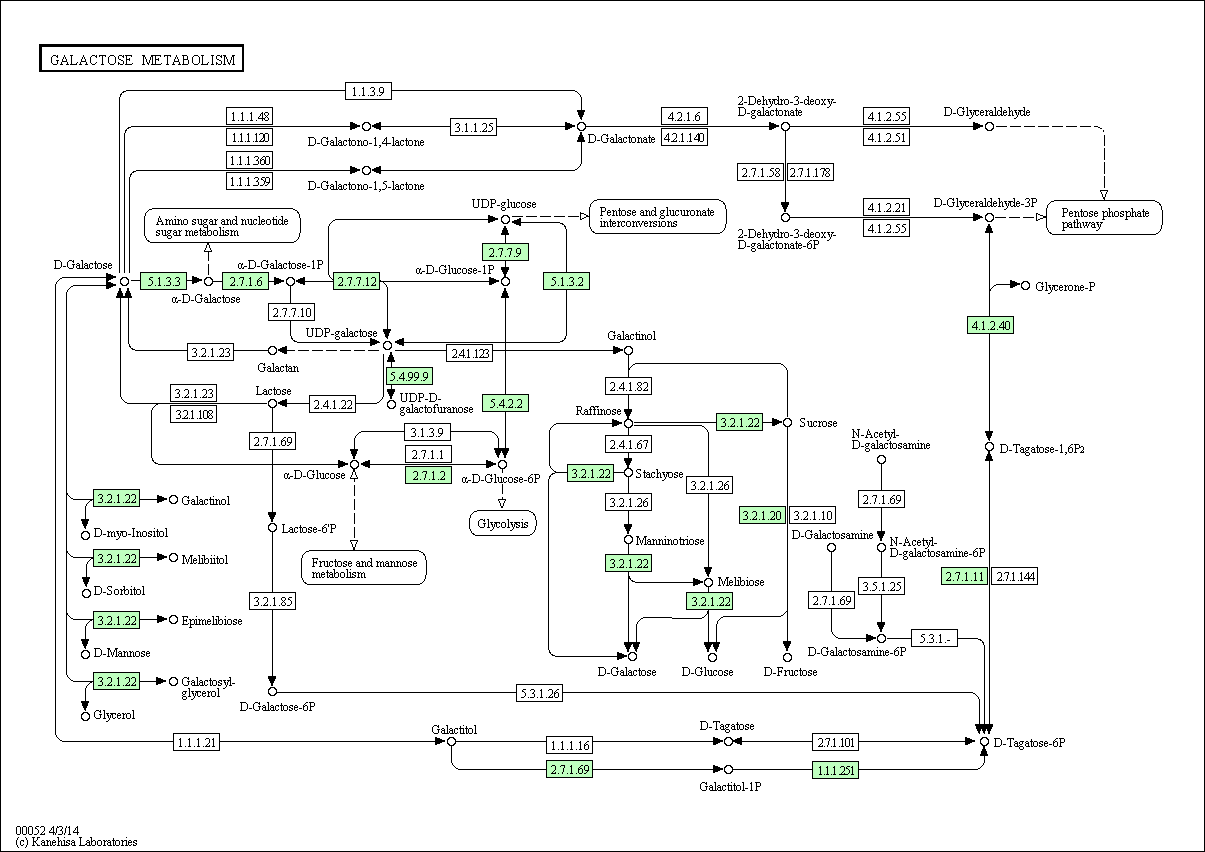

Supplement: S1 File — (ZIP) [file pone.0126207.s001.zip › S1 File/Galactose Metabolism.png]

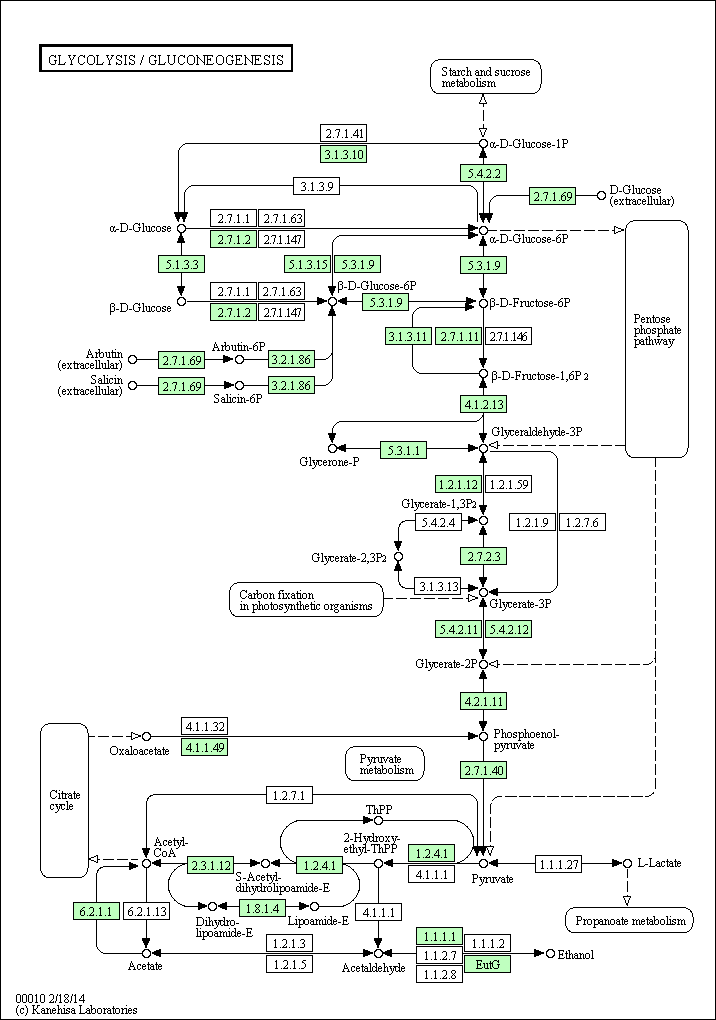

Supplement: S1 File — (ZIP) [file pone.0126207.s001.zip › S1 File/Glycolysis_Gluconeogenesis.png]

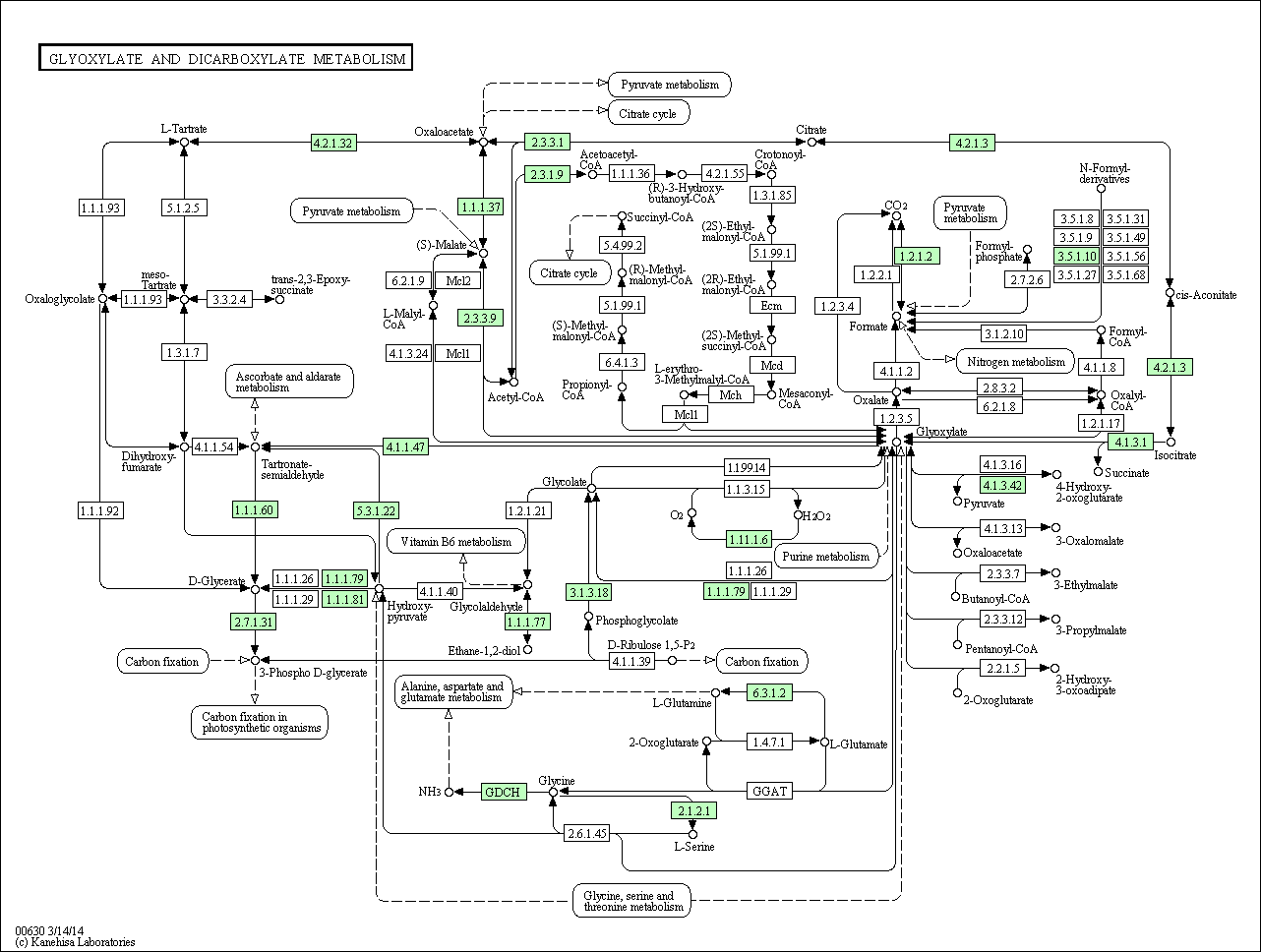

Supplement: S1 File — (ZIP) [file pone.0126207.s001.zip › S1 File/Glyoxylate and Dicarboxylate Metabolism.png]

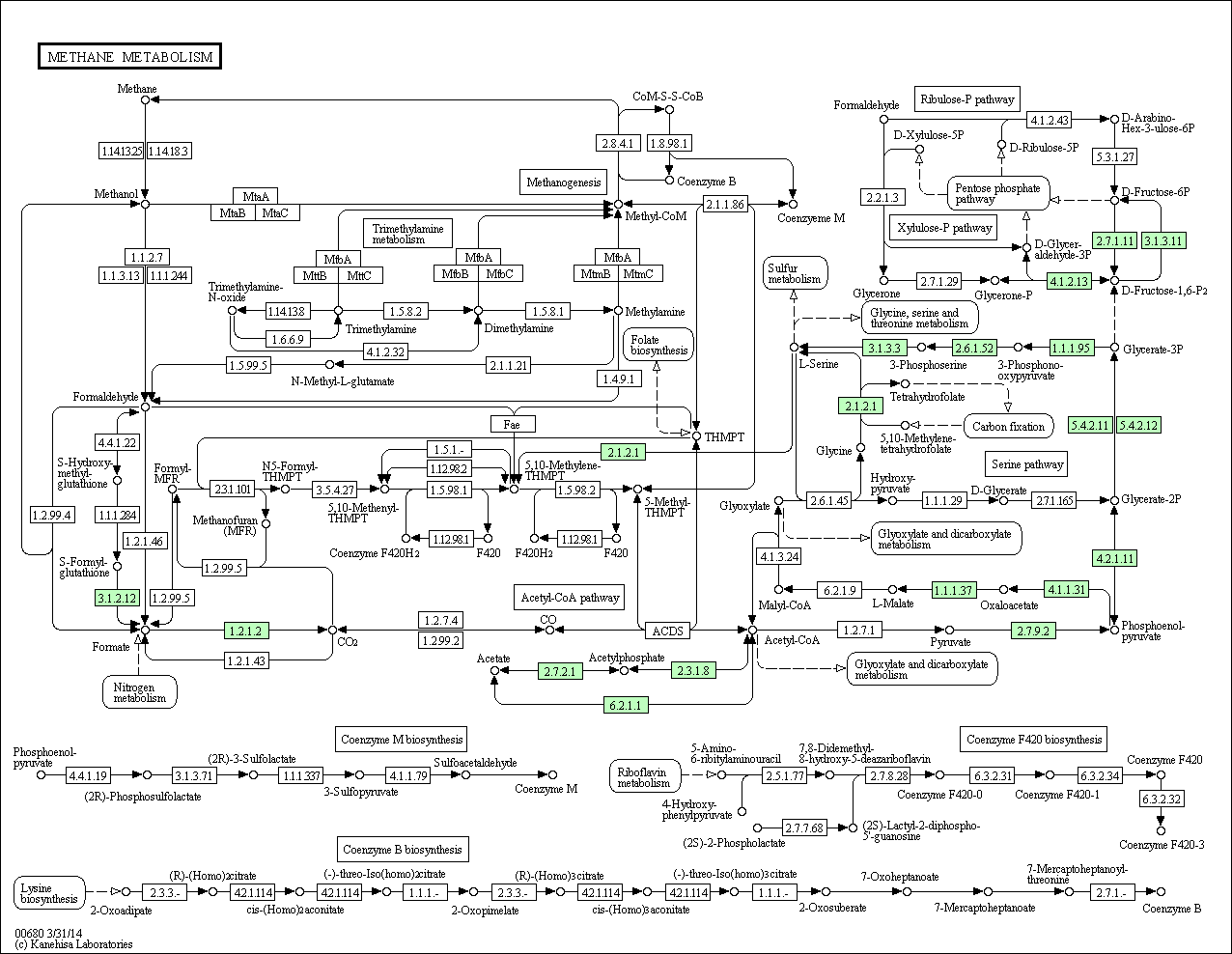

Supplement: S1 File — (ZIP) [file pone.0126207.s001.zip › S1 File/Methane Metabolism.png]

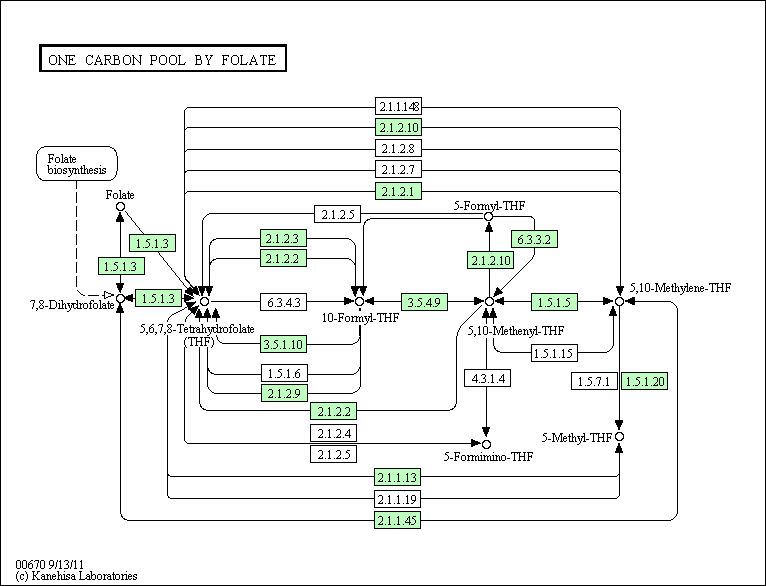

Supplement: S1 File — (ZIP) [file pone.0126207.s001.zip › S1 File/One Carbon Pool by Folate.png]

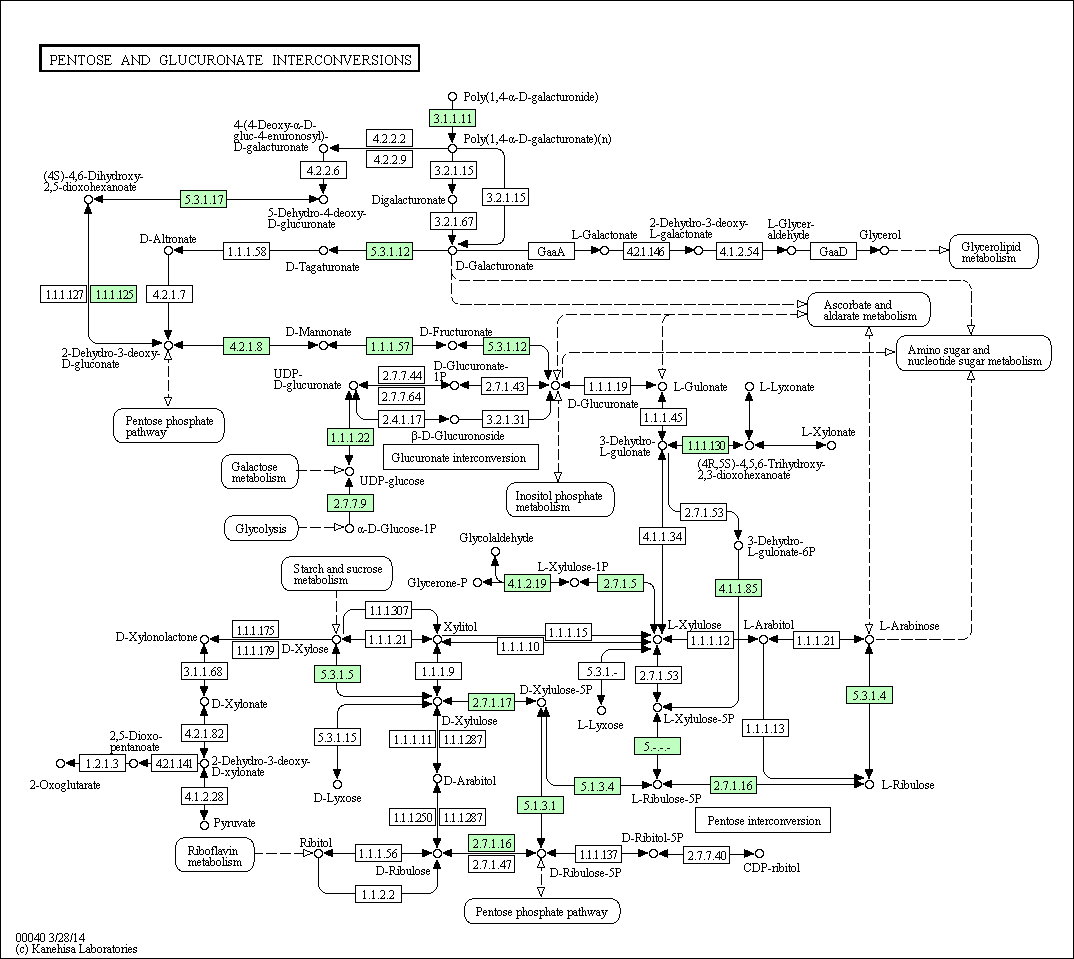

Supplement: S1 File — (ZIP) [file pone.0126207.s001.zip › S1 File/Pentose and Glucoronate Interconversions.png]

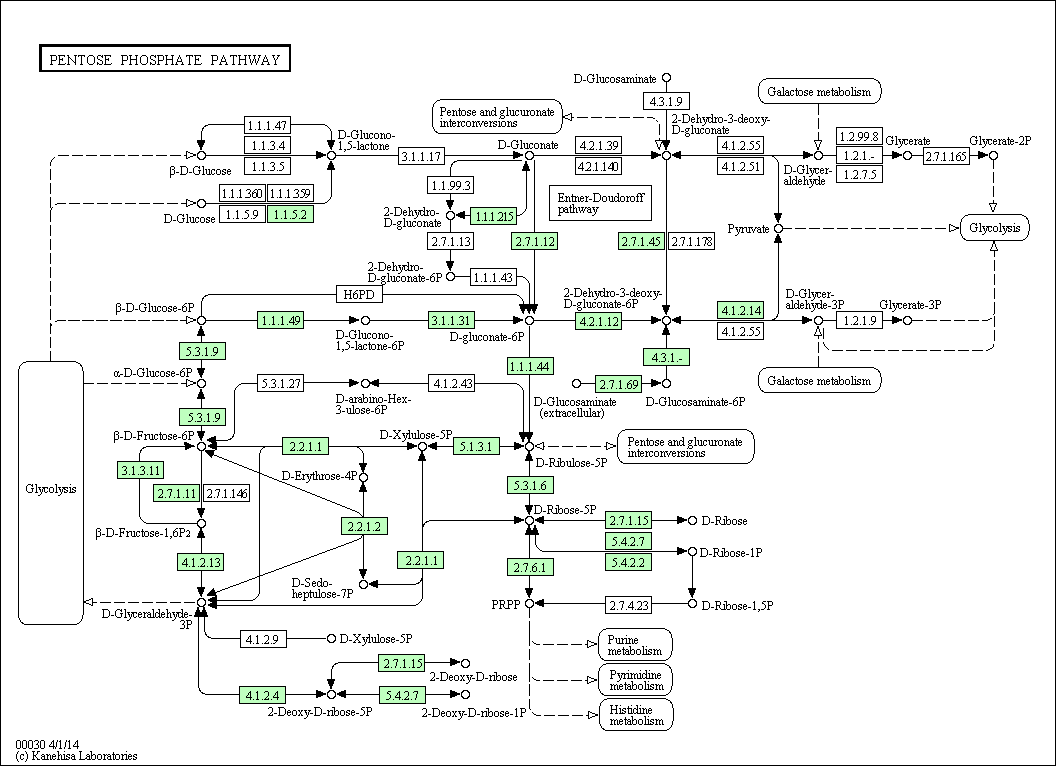

Supplement: S1 File — (ZIP) [file pone.0126207.s001.zip › S1 File/Pentose Phosphate Pathway.png]

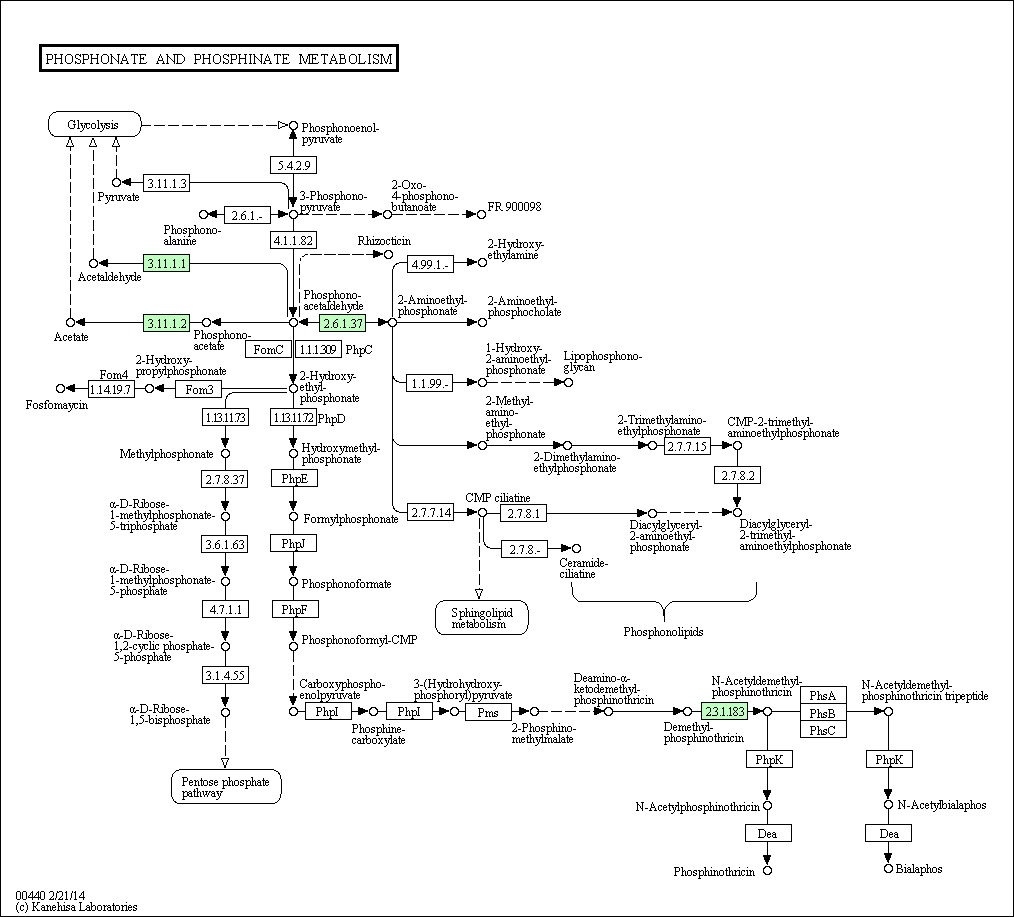

Supplement: S1 File — (ZIP) [file pone.0126207.s001.zip › S1 File/Phosphonate and Phosphinate Metabolism.png]

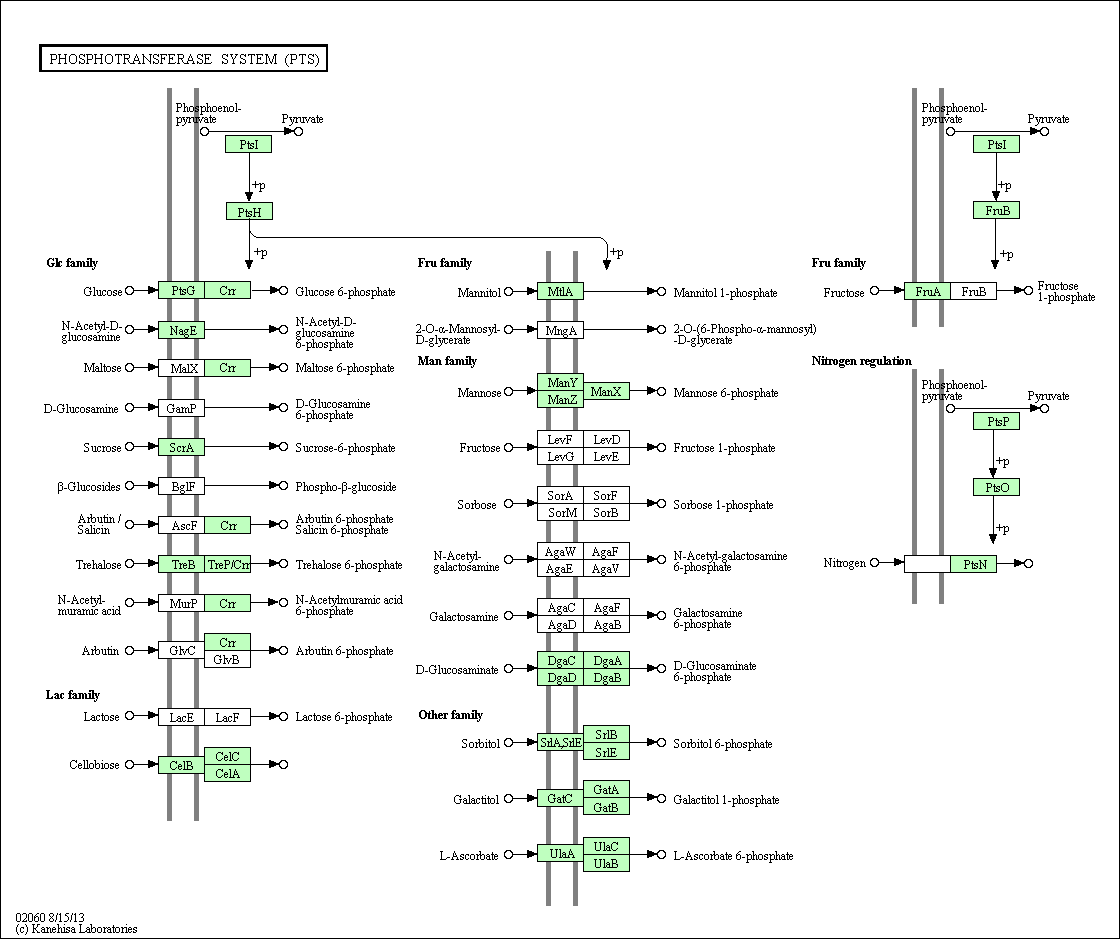

Supplement: S1 File — (ZIP) [file pone.0126207.s001.zip › S1 File/Phosphotransferase System (PTS).png]

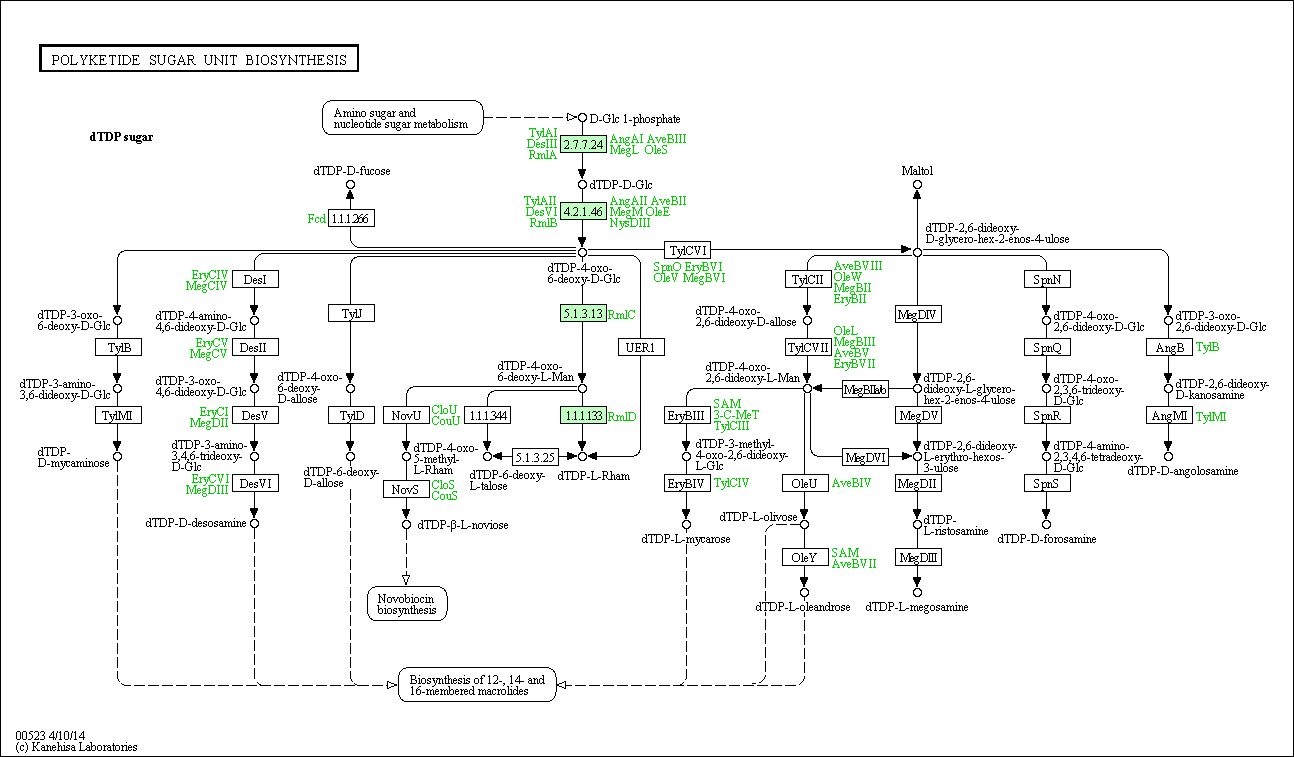

Supplement: S1 File — (ZIP) [file pone.0126207.s001.zip › S1 File/Polyketide Sugar Unit Biosynthesis.png]

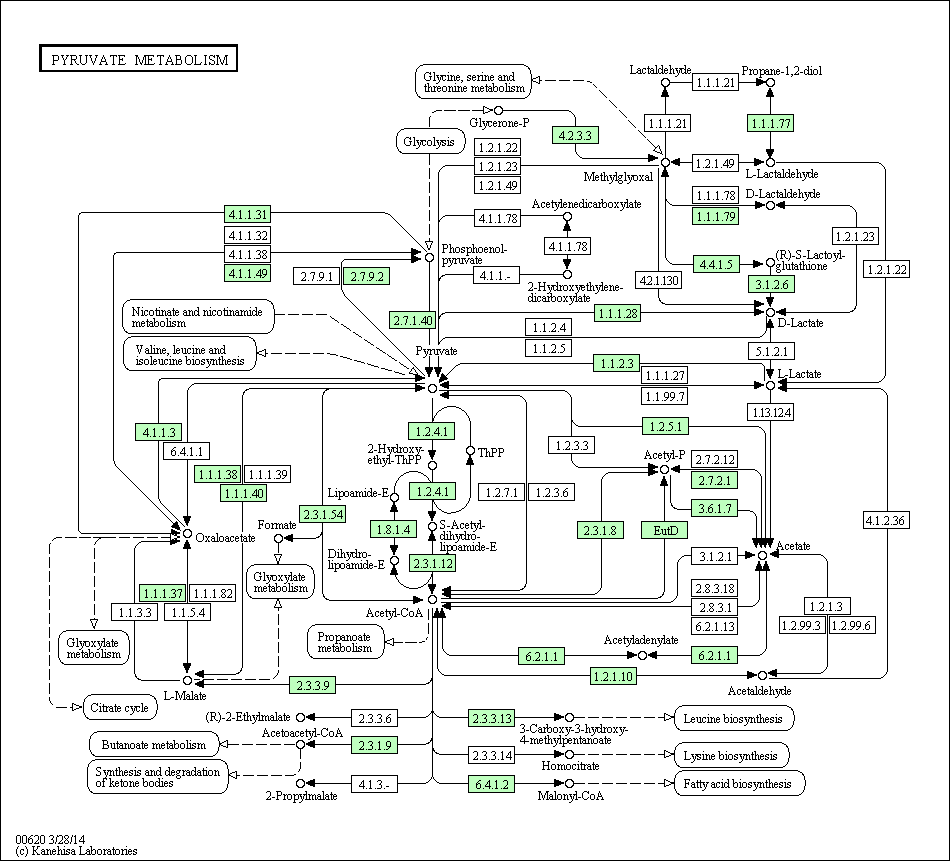

Supplement: S1 File — (ZIP) [file pone.0126207.s001.zip › S1 File/Pyruvate Metabolism.png]

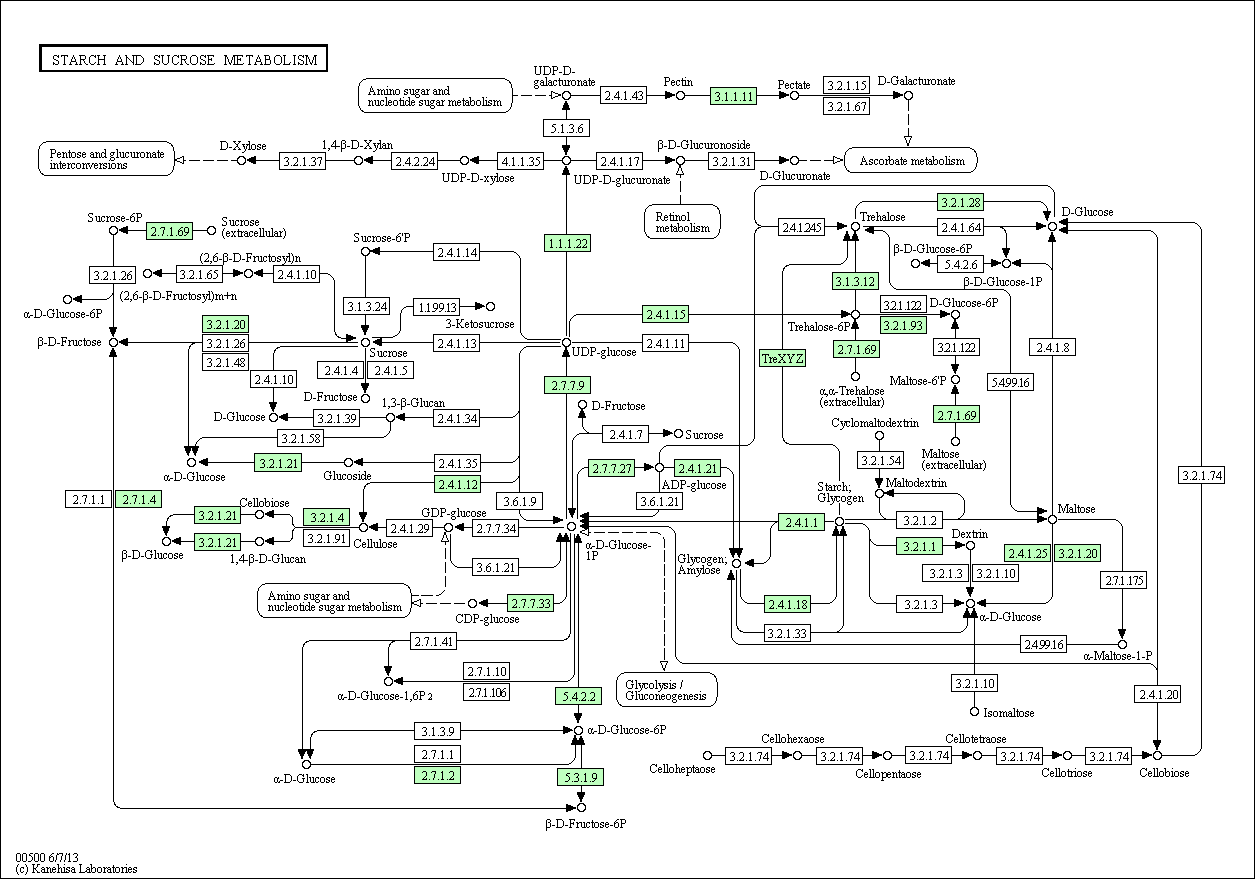

Supplement: S1 File — (ZIP) [file pone.0126207.s001.zip › S1 File/Sucrose metabolism.png]

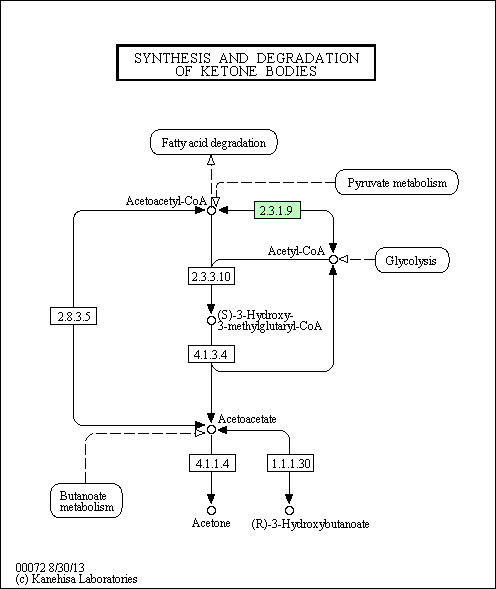

Supplement: S1 File — (ZIP) [file pone.0126207.s001.zip › S1 File/Synthesis and Degradation of Ketone bodies.png]
