# Supplementary material for: Variable Responses to Carbon Utilization between Planktonic and Biofilm Cells of a Human Carrier Strain of Salmonella enterica Serovar Typhi
Source: PLoS One. 2015 May 6;10(5):e0126207. doi: 10.1371/journal.pone.0126207 (PMC4422432; doi:10.1371/journal.pone.0126207)
Supplement: S1 Table — (PDF) [file pone.0126207.s002.pdf]

S1 Table. *Salmonella enterica* serovar Typhi strains used in this study.

Strain	Year	Source	Locality	Biofilm	Motility
CR0044	2007	Healthy human carrier (stool)	Kelantan, Malaysia	Strong	+
BL191	2005	Human (blood); outbreak	Kelantan, Malaysia	Strong	+++
BL196	2005	Human (blood); outbreak	Kelantan, Malaysia	Strong	+++
S5680	2007	Human (stool); outbreak	Kelantan, Malaysia	Strong	+++
ST33	2006	Human (blood); outbreak	Kelantan, Malaysia	Weak	+++
ST280	1990	Human (stool); outbreak	Johor, Malaysia	Moderate	++
STVC1681	1983	Sewage contaminated water	Chile	Moderate	+++
STVC3121	1983	Human (blood); outbreak	Chile	Moderate	+++
