# Supplementary material for: Variable Responses to Carbon Utilization between Planktonic and Biofilm Cells of a Human Carrier Strain of Salmonella enterica Serovar Typhi
Source: PLoS One. 2015 May 6;10(5):e0126207. doi: 10.1371/journal.pone.0126207 (PMC4422432; doi:10.1371/journal.pone.0126207)
Supplement: S2 Table — (PDF) [file pone.0126207.s003.pdf]

S2 Table. **List of all the conditions/ substrates in PM wells for plates PM1 and PM2A**

Plate	Location	Chemical	CAS ID	KEGG ID	MoA
PM01	A01	Negative Control			C-Source, negative control
PM01	A02	L-Arabinose	CAS 87-72-9	C00259	C-Source, carbohydrate
PM01	A03	N-Acetyl-D-Glucosamine	CAS 7512-17-6	C00140	C-Source, carbohydrate
PM01	A04	D-Saccharic acid	CAS 576-42-1	C00818	C-Source, carboxylic acid
PM01	A05	Succinic acid	CAS 6106-21-4	C00042	C-Source, carboxylic acid
PM01	A06	D-Galactose	CAS 59-23-4	C00124	C-Source, carbohydrate
PM01	A07	L-Aspartic acid	CAS 3792-50-5	C00049	C-Source, amino acid
PM01	A08	L-Proline	CAS 147-85-3	C00148	C-Source, amino acid
PM01	A09	D-Alanine	CAS 338-69-2	C00133	C-Source, amino acid
PM01	A10	D-Trehalose	CAS 6138-23-4	C01083	C-Source, carbohydrate
PM01	A11	D-Mannose	CAS 3458-28-4	C00159	C-Source, carbohydrate
PM01	A12	Dulcitol	CAS 608-66-2	C01697	C-Source, carbohydrate
PM01	B01	D-Serine	CAS 312-84-5	C00740	C-Source, amino acid
PM01	B02	D-Sorbitol	CAS 50-70-4	C00794	C-Source, carbohydrate
PM01	B03	Glycerol	CAS 56-81-5	C00116	C-Source, carbohydrate
PM01	B04	L-Fucose	CAS 2438-80-4	C01019	C-Source, carbohydrate
PM01	B05	D-Glucuronic acid	CAS 14984-34-0	C00191	C-Source, carboxylic acid
PM01	B06	D-Gluconic acid	CAS 527-07-1	C00257	C-Source, carboxylic acid
PM01	B07	DL-a-Glycerol Phosphate	CAS 3325-00-6	C00093	C-Source, carbohydrate
PM01	B08	D-Xylose	CAS 58-86-6	C00181	C-Source, carbohydrate
PM01	B09	L-Lactic acid	CAS 312-85-6	C01432	C-Source, carboxylic acid
PM01	B10	Formic acid	CAS 141-53-7	C00058	C-Source, carboxylic acid

Plate	Location	Chemical	CAS ID	KEGG ID	MoA
PM01	B11	D-Mannitol	CAS 69-65-8	C00392	C-Source, carbohydrate
PM01	B12	L-Glutamic acid	CAS 6106-04-3	C00025	C-Source, amino acid
PM01	C01	D-Glucose-6-Phosphate	CAS 3671-99-6	C00092	C-Source, carbohydrate
PM01	C02	D-Galactonic acid-g-Lactone	CAS 2782-07-2	C03383	C-Source, carboxylic acid
PM01	C03	DL-Malic acid	CAS 6915-15-7	C00497	C-Source, carboxylic acid
PM01	C04	D-Ribose	CAS 50-69-1	C00121	C-Source, carbohydrate
PM01	C05	Tween 20	CAS 9005-64-5	C11624	C-Source, fatty acid
PM01	C06	L-Rhamnose	CAS 3615-41-6	C00507	C-Source, carbohydrate
PM01	C07	D-Fructose	CAS 57-48-7	C00095	C-Source, carbohydrate
PM01	C08	Acetic acid	CAS 127-09-3	C00033	C-Source, carboxylic acid
PM01	C09	a-D-Glucose	CAS 50-99-7	C00031	C-Source, carbohydrate
PM01	C10	Maltose	CAS 6363-53-7	C00208	C-Source, carbohydrate
PM01	C11	D-Melibiose	CAS 585-99-9	C05402	C-Source, carbohydrate
PM01	C12	Thymidine	CAS 50-89-5	C00214	C-Source, carbohydrate
PM01	D01	L-Asparagine	CAS 70-47-3	C00152	C-Source, amino acid
PM01	D02	D-Aspartic acid	CAS 1783-96-6	C00402	C-Source, amino acid
PM01	D03	D-Glucosaminic acid	CAS 3646-68-2	C03752	C-Source, carboxylic acid
PM01	D04	1,2-Propanediol	CAS 57-55-6	C00583	C-Source, alcohol
PM01	D05	Tween 40	CAS 9005-66-7		C-Source, fatty acid
PM01	D06	a-Ketoglutaric acid	CAS 22202-68-2	C00026	C-Source, carboxylic acid
PM01	D07	a-Ketobutyric acid	CAS 2013-26-5	C00109	C-Source, carboxylic acid
PM01	D08	a-Methyl-D-Galactoside	CAS 3396-99-4	C03619	C-Source, carbohydrate
PM01	D09	a-D-Lactose	CAS 5989-81-1	C00243	C-Source, carbohydrate
PM01	D10	Lactulose	CAS 4618-18-2	C07064	C-Source, carbohydrate

Plate	Location	Chemical	CAS ID	KEGG ID	MoA
PM01	D11	Sucrose	CAS 57-50-1	C00089	C-Source, carbohydrate
PM01	D12	Uridine	CAS 58-96-8	C00299	C-Source, carbohydrate
PM01	E01	L-Glutamine	CAS 56-85-9	C00064	C-Source, amino acid
PM01	E02	m-Tartaric acid	CAS 147-73-9	C00552	C-Source, carboxylic acid
PM01	E03	D-Glucose-1-Phosphate	CAS 56401-20-8	C00103	C-Source, carbohydrate
PM01	E04	D-Fructose-6-Phosphate	CAS 26177-86-6	C00085	C-Source, carbohydrate
PM01	E05	Tween 80	CAS 9005-65-6	C11625	C-Source, fatty acid
PM01	E06	a-Hydroxyglutaric acid-g-Lactone	CAS 21461-84-7		C-Source, carboxylic acid
PM01	E07	a-Hydroxybutyric acid	CAS 19054-57-0	C05984	C-Source, carboxylic acid
PM01	E08	b-Methyl-D-Glucoside	CAS 709-50-2		C-Source, carbohydrate
PM01	E09	Adonitol	CAS 488-81-3	C00474	C-Source, carbohydrate
PM01	E10	Maltotriose	CAS 1109-28-0	C01835	C-Source, carbohydrate
PM01	E11	2`-Deoxyadenosine	CAS 16373-93-6	C00559	C-Source, carbohydrate
PM01	E12	Adenosine	CAS 58-61-7	C00212	C-Source, carbohydrate
PM01	F01	Gly-Asp	CAS 4685-12-5	C02871	C-Source, amino acid
PM01	F02	Citric acid	CAS 6132-04-3	C00158	C-Source, carboxylic acid
PM01	F03	m-Inositol	CAS 87-89-8	C00137	C-Source, carbohydrate
PM01	F04	D-Threonine	CAS 632-20-2	C00820	C-Source, amino acid
PM01	F05	Fumaric acid	CAS 17013-01-3	C00122	C-Source, carboxylic acid
PM01	F06	Bromosuccinic acid	CAS 923-06-8		C-Source, carboxylic acid
PM01	F07	Propionic acid	CAS 137-40-6	C00163	C-Source, carboxylic acid
PM01	F08	Mucic acid	CAS 526-99-8	C01807	C-Source, carboxylic acid
PM01	F09	Glycolic acid	CAS 79-14-1	C00160	C-Source, carboxylic acid
PM01	F10	Glyoxylic acid	CAS 563-96-2	C00048	C-Source, carboxylic acid
PM01	F11	D-Cellobiose	CAS 528-50-7	C00185	C-Source, carbohydrate
PM01	F12	Inosine	CAS 58-63-9	C00294	C-Source, carbohydrate
PM01	G01	Gly-Glu	CAS 7412-78-4		C-Source, amino acid

Plate	Location	Chemical	CAS ID	KEGG ID	MoA
PM01	G02	Tricarballylic acid	CAS 99-14-9		C-Source, carboxylic acid
PM01	G03	L-Serine	CAS 56-45-1	C00065	C-Source, amino acid
PM01	G04	L-Threonine	CAS 72-19-5	C00188	C-Source, amino acid
PM01	G05	L-Alanine	CAS 56-41-7	C00041	C-Source, amino acid
PM01	G06	Ala-Gly	CAS 687-69-4		C-Source, amino acid
PM01	G07	Acetoacetic acid	CAS 3483-11-2	C00164	C-Source, carboxylic acid
PM01	G08	N-Acetyl-D-Mannosamine	CAS 7772-94-3	C00645	C-Source, carbohydrate
PM01	G09	Mono-Methylsuccinate	CAS 3878-55-5		C-Source, carboxylic acid
PM01	G10	Methylpyruvate	CAS 600-22-6		C-Source, ester
PM01	G11	D-Malic acid	CAS 636-61-3	C00497	C-Source, carboxylic acid
PM01	G12	L-Malic acid	CAS 138-09-0	C00149	C-Source, carboxylic acid
PM01	H01	Gly-Pro	CAS 704-15-4		C-Source, amino acid
PM01	H02	p-Hydroxyphenyl Acetic acid	CAS 156-38-7	C00642	C-Source, carboxylic acid
PM01	H03	m-Hydroxyphenyl Acetic acid	CAS 621-37-4	C05593	C-Source, carboxylic acid
PM01	H04	Tyramine	CAS 60-19-5	C00483	C-Source, amine
PM01	H05	D-Psicose	CAS 551-68-8	C06468	C-Source, carbohydrate
PM01	H06	L-Lyxose	CAS 1949-78-6	C01508	C-Source, carbohydrate
PM01	H07	Glucuronamide	CAS 3789-97-7	D01791	C-Source, amide
PM01	H08	Pyruvic acid	CAS 113-24-6	C00022	C-Source, carboxylic acid
PM01	H09	L-Galactonic acid-g-Lactone	CAS 1668-08-2	C01115	C-Source, carboxylic acid
PM01	H10	D-Galacturonic acid	CAS 91510-62-2	C00333	C-Source, carboxylic acid
PM01	H11	Phenylethylamine	CAS 156-28-5	C05332	C-Source, amine
PM01	H12	2-Aminoethanol	CAS 141-43-5	C00189	C-Source, alcohol
PM02A	A01	Negative Control			C-Source, negative control
PM02A	A02	Chondroitin Sulfate C	CAS 12678-07-8	C00635	C-Source, polymer
PM02A	A03	a-Cyclodextrin	CAS 10016-20-3		C-Source, polymer

Plate	Location	Chemical	CAS ID	KEGG ID	MoA
PM02A	A04	b-Cyclodextrin	CAS 7585-39-9		C-Source, polymer
PM02A	A05	g-Cyclodextrin	CAS 17465-86-0		C-Source, polymer
PM02A	A06	Dextrin	CAS 9004-53-9	C00721	C-Source, polymer
PM02A	A07	Gelatin	CAS 9000-70-8	C01498	C-Source, polymer
PM02A	A08	Glycogen	CAS 9005-79-2	C00182	C-Source, polymer
PM02A	A09	Inulin	CAS 9005-80-5	C00368	C-Source, polymer
PM02A	A10	Laminarin	CAS 9008-22-4	C00771	C-Source, polymer
PM02A	A11	Mannan	CAS 9036-88-8	C00464	C-Source, polymer
PM02A	A12	Pectin	CAS 9000-69-5	C00714	C-Source, polymer
PM02A	B01	N-Acetyl-D-Galactosamine	CAS 14215-68-0	C01074	C-Source, carbohydrate
PM02A	B02	N-Acetyl-Neuraminic acid	CAS 131-48-6	C00270	C-Source, carboxylic acid
PM02A	B03	b-D-Allose	CAS 2595-97-3	C01487	C-Source, carbohydrate
PM02A	B04	Amygdalin	CAS 29883-15-6	C08325	C-Source, carbohydrate
PM02A	B05	D-Arabinose	CAS 10323-20-3	C00216	C-Source, carbohydrate
PM02A	B06	D-Arabitol	CAS 488-82-4	C01904	C-Source, carbohydrate
PM02A	B07	L-Arabitol	CAS 7643-75-6	C00532	C-Source, carbohydrate
PM02A	B08	Arbutin	CAS 497-76-7	C06186	C-Source, carbohydrate
PM02A	B09	2-Deoxy-D-Ribose	CAS 533-67-5	C01801	C-Source, carbohydrate
PM02A	B10	i-Erythritol	CAS 149-32-6	C00503	C-Source, carbohydrate
PM02A	B11	D-Fucose	CAS 3615-37-0	C01018	C-Source, carbohydrate
PM02A	B12	3-O-b-D-Galactopyranosyl-D-Arabinose	CAS 6057-48-3		C-Source, carbohydrate
PM02A	C01	Gentiobiose	CAS 554-91-6	C08240	C-Source, carbohydrate
PM02A	C02	L-Glucose	CAS 921-60-8		C-Source, carbohydrate
PM02A	C03	D-Lactitol	CAS 81025-04-9		C-Source, carbohydrate
PM02A	C04	D-Melezitose	CAS 10030-67-8	C08243	C-Source, carbohydrate
PM02A	C05	Maltitol	CAS 585-88-6	G00275	C-Source, carbohydrate
PM02A	C06	a-Methyl-D-Glucoside	CAS 97-30-3		C-Source, carbohydrate

Plate	Location	Chemical	CAS ID	KEGG ID	MoA
PM02A	C07	b-Methyl-D-Galactoside	CAS 1824-94-8	C03619	C-Source, carbohydrate
PM02A	C08	3-Methylglucose	CAS 13224-94-7		C-Source, carbohydrate
PM02A	C09	b-Methyl-D-Glucuronic acid	CAS 134253-42-2	C08350	C-Source, carboxylic acid
PM02A	C10	a-Methyl-D-Mannoside	CAS 617-04-9		C-Source, carbohydrate
PM02A	C11	b-Methyl-D-Xyloside	CAS 612-05-5		C-Source, carbohydrate
PM02A	C12	Palatinose	CAS 13718-94-0	C01742	C-Source, carbohydrate
PM02A	D01	D-Raffinose	CAS 17629-30-0	C00492	C-Source, carbohydrate
PM02A	D02	Salicin	CAS 138-52-3	C01451	C-Source, carbohydrate
PM02A	D03	Sedoheptulosan	CAS 469-90-9		C-Source, carbohydrate
PM02A	D04	L-Sorbose	CAS 87-79-6	C00247	C-Source, carbohydrate
PM02A	D05	Stachyose	CAS 54261-98-2	C01613	C-Source, carbohydrate
PM02A	D06	D-Tagatose	CAS 87-81-0	C00795	C-Source, carbohydrate
PM02A	D07	Turanose	CAS 547-25-1	G03588	C-Source, carbohydrate
PM02A	D08	Xylitol	CAS 87-99-0	C00379	C-Source, carbohydrate
PM02A	D09	N-Acetyl-D-Glucosaminitol	CAS 4271-28-7		C-Source, carbohydrate
PM02A	D10	g-Amino-N-Butyric acid	CAS 56-12-2	C00334	C-Source, carboxylic acid
PM02A	D11	d-Amino Valeric acid	CAS 5451-09-2	C00431	C-Source, carboxylic acid
PM02A	D12	Butyric acid	CAS 156-54-7	C00246	C-Source, carboxylic acid
PM02A	E01	Capric acid	CAS 1002-62-6	C01571	C-Source, carboxylic acid
PM02A	E02	Caproic acid	CAS 10051-44-2	C01585	C-Source, carboxylic acid
PM02A	E03	Citraconic acid	CAS 207-858-7	C02226	C-Source, carboxylic acid
PM02A	E04	Citramalic acid	CAS 6236-10-8	C00815	C-Source, carboxylic acid
PM02A	E05	D-Glucosamine	CAS 66-84-2	C00329	C-Source, carbohydrate
PM02A	E06	2-Hydroxybenzoic acid	CAS 54-21-7	C00805	C-Source, carboxylic acid
PM02A	E07	4-Hydroxybenzoic acid	CAS 114-63-6	C00156	C-Source, carboxylic acid
PM02A	E08	b-Hydroxybutyric acid	CAS 150-83-4	C01089	C-Source, carboxylic acid

Plate	Location	Chemical	CAS ID	KEGG ID	MoA
PM02A	E09	g-Hydroxybutyric acid	CAS 502-85-2	C00989	C-Source, carboxylic acid
PM02A	E10	a-Keto-Valeric acid	CAS 1821-02-9	C00567	C-Source, carboxylic acid
PM02A	E11	Itaconic acid	CAS 97-65-4	C00490	C-Source, carboxylic acid
PM02A	E12	5-Keto-D-Gluconic acid	CAS 91446-96-7	C01062	C-Source, carboxylic acid
PM02A	F01	D-Lactic acid Methyl Ester	CAS 17392-83-5		C-Source, ester
PM02A	F02	Malonic acid	CAS 26522-85-0	C00383	C-Source, carboxylic acid
PM02A	F03	Melibionic acid	CAS 70803-54-2		C-Source, carbohydrate
PM02A	F04	Oxalic acid	CAS 62-76-0	C00209	C-Source, carboxylic acid
PM02A	F05	Oxalomalic acid	CAS 89304-26-7	C01990	C-Source, carboxylic acid
PM02A	F06	Quinic acid	CAS 77-95-2	C00296	C-Source, carboxylic acid
PM02A	F07	D-Ribono-1,4-Lactone	CAS 5336-08-3		C-Source, carboxylic acid
PM02A	F08	Sebacic acid	CAS 111-20-6	C08277	C-Source, carboxylic acid
PM02A	F09	Sorbic acid	CAS 110-44-1		C-Source, carboxylic acid
PM02A	F10	Succinamic acid	CAS 638-32-4		C-Source, carboxylic acid
PM02A	F11	D-Tartaric acid	CAS 57341-16-9	C02107	C-Source, carboxylic acid
PM02A	F12	L-Tartaric acid	CAS 6106-24-7	C00898	C-Source, carboxylic acid
PM02A	G01	Acetamide	CAS 60-35-5	C06244	C-Source, amide
PM02A	G02	L-Alaninamide	CAS 33208-99-0		C-Source, amide
PM02A	G03	N-Acetyl-L-Glutamic acid	CAS 1188-37-0	C00624	C-Source, amino acid
PM02A	G04	L-Arginine	CAS 1119-34-2	C00062	C-Source, amino acid
PM02A	G05	Glycine	CAS 6000-43-7	C00037	C-Source, amino acid
PM02A	G06	L-Histidine	CAS 5934-29-2	C00135	C-Source, amino acid
PM02A	G07	L-Homoserine	CAS 672-15-1	C00263	C-Source, amino acid
PM02A	G08	Hydroxy-L-Proline	CAS 51-35-4	C01015	C-Source, amino acid
PM02A	G09	L-Isoleucine	CAS 73-32-5	C00407	C-Source, amino acid
PM02A	G10	L-Leucine	CAS 61-90-5	C00123	C-Source, amino acid

Plate	Location	Chemical	CAS ID	KEGG ID	MoA
PM02A	G11	L-Lysine	CAS 657-27-2	C00047	C-Source, amino acid
PM02A	G12	L-Methionine	CAS 63-68-3	C00073	C-Source, amino acid
PM02A	H01	L-Ornithine	CAS 3184-13-2	C00077	C-Source, amino acid
PM02A	H02	L-Phenylalanine	CAS 63-91-2	C00079	C-Source, amino acid
PM02A	H03	L-Pyroglutamic acid	CAS 98-79-3	C02238	C-Source, amino acid
PM02A	H04	L-Valine	CAS 72-18-4	C00183	C-Source, amino acid
PM02A	H05	D,L-Carnitine	CAS 461-05-2	C00487	C-Source, carboxylic acid
PM02A	H06	sec-Butylamine	CAS 13952-84-6		C-Source, amine
PM02A	H07	D,L-Octopamine	CAS 770-05-8	C04227	C-Source, amine
PM02A	H08	Putrescine	CAS 333-93-7	C00134	C-Source, amine
PM02A	H09	Dihydroxyacetone	CAS 96-26-4	C00184	C-Source, alcohol
PM02A	H10	2,3-Butanediol	CAS 513-85-9	C03044	C-Source, alcohol
PM02A	H11	2,3-Butanedione	CAS 431-03-8	C00741	C-Source, alcohol
PM02A	H12	3-Hydroxy-2-butanone	CAS 513-86-0	C00466	C-Source, alcohol
