# Supplementary material for: Variable Responses to Carbon Utilization between Planktonic and Biofilm Cells of a Human Carrier Strain of Salmonella enterica Serovar Typhi
Source: PLoS One. 2015 May 6;10(5):e0126207. doi: 10.1371/journal.pone.0126207 (PMC4422432; doi:10.1371/journal.pone.0126207)
Supplement: S3 Table — (PDF) [file pone.0126207.s004.pdf]

S3 Table. Raw Phenotype MicroArray data and presence/ absence of catabolism- related genes in the *S*. Typhi human carrier strain CR0044.

Code	Carbon	Group	Planktonic only	Biofilm only	Planktonic and Biofilm only	Not catabolised	Induce biofilm	Gene	Minimal Media Result
PM1_D4	1,2-Propanediol	alcohol	0	0	0	1	2	Partial	
PM1_H12	2-Aminoethanol	alcohol	0	0	0	1	1	Complete	
PM2_H10	2,3-Butanediol	alcohol	0	0	0	1	[illegible]	Absent	
PM2_H11	2,3-Butanone	alcohol	0	0	0	1	[illegible]	Absent	
PM2_H12	3-Hydroxy-2-butanone	alcohol	0	0	0	1	[illegible]	Absent	
PM2_H9	Dihydroxyacetone	alcohol	0	0	0	1	[illegible]	Complete	
PM1_H7	Glucuronamide	amide	0	0	0	1	1	Absent	
PM2_G1	Acetamide	amide	0	0	0	1	4	Absent	
PM2_G2	L-Alaninamide	amide	0	0	0	1	5	Absent	
PM1_H11	Phenylethylamine	amine	0	0	0	1	1	Absent	
PM1_H4	Tyramine	amine	0	0	0	1	2	Absent	
PM2_H6	sec-Butylamine	amine	0	0	0	1	[illegible]	Absent	
PM2_H7	D,L-Octopamine	amine	0	0	0	1	[illegible]	Absent	
PM2_H8	Putrescine	amine	0	0	0	1	[illegible]	Complete	
PM1_A7	L-Aspartic acid	amino acid	0	0	1	0	2	Partial	
PM1_A8	L-Proline	amino acid	0	0	1	0	2	Complete	
PM1_A9	D-Alanine	amino acid	1	0	0	0	2	Complete	
PM1_B1	D-Serine	amino acid	1	0	0	0	1	Complete	
PM1_B12	L-Glutamic acid	amino acid	0	0	1	0	1	Partial	
PM1_D1	L-Asparagine	amino acid	0	0	1	0	2	Complete	
PM1_D2	D-Aspartic acid	amino acid	0	0	0	1	2	Partial	
PM1_E1	L-Glutamine	amino acid	1	0	0	0	2	Complete	
PM1_F1	Gly-Asp	amino acid	1	0	0	0	2	Absent	
PM1_F4	D-Threonine	amino acid	0	1	0	0	2	Partial	
PM1_G1	Gly-Glu	amino acid	1	0	0	0	2	Absent	
PM1_G3	L-Serine	amino acid	0	0	1	0	2	Partial	
PM1_G4	L-Threonine	amino acid	0	0	1	0	2	Complete	
PM1_G5	L-Alanine	amino acid	1	0	0	0	2	Complete	
PM1_G6	Ala-Gly	amino acid	1	0	0	0	2	Absent	
PM1_H1	Gly-Pro	amino acid	0	0	1	0	2	Absent	
PM2_G10	L-Leucine	amino acid	0	0	0	1	[illegible]	Complete	
PM2_G11	L-Lysine	amino acid	0	0	0	1	4	Absent	
PM2_G12	L-Methionine	amino acid	0	0	0	1	4	Absent	
PM2_G3	N-Acetyl-L-Glutamic acid	amino acid	0	0	0	1	4	Complete	
PM2_G4	L-Arginine	amino acid	0	0	0	1	5	Absent	
PM2_G5	Glycine	amino acid	0	0	0	1	5	Complete	
PM2_G6	L-Histidine	amino acid	0	0	0	1	3	Complete	
PM2_G7	L-Homoserine	amino acid	0	0	0	1	3	Complete	
PM2_G8	Hydroxy-L-Proline	amino acid	0	0	0	1	5	Absent	
PM2_G9	L-Isoleucine	amino acid	0	0	0	1	[illegible]	Complete	
PM2_H1	L-Ornithine	amino acid	0	0	0	1	4	Absent	
PM2_H2	L-Phenylalanine	amino acid	0	0	0	1	5	Complete	
PM2_H3	L-Pyroglutamic acid	amino acid	0	0	0	1	[illegible]	Absent	
PM2_H4	L-Valine	amino acid	0	0	0	1	4	Complete	
PM1_A10	D-Trehalose	carbohydrate	0	0	1	0	2	Complete	
PM1_A11	D-Mannose	carbohydrate	1	0	0	0	2	Partial	
PM1_A12	Dulcitol	carbohydrate	0	0	0	1	2	Complete	
PM1_A2	L-Arabinose	carbohydrate	0	0	0	1	1	Partial	

Code	Carbon	Group	Planktonic only	Biofilm only	Planktonic and Biofilm only	Not catabolised	Induce biofilm	Gene	Minimal Media Result
PM1_A3	N-Acetyl-D-Glucosamine	carbohydrate	0	0	1	0	2	Complete	
PM1_A6	D-Galactose	carbohydrate	1	0	0	0	1	Complete	
PM1_B11	D-Mannitol	carbohydrate	0	0	1	0	1	Complete	
PM1_B2	D-Sorbitol	carbohydrate	0	0	1	0	2	Complete	
PM1_B3	Glycerol	carbohydrate	0	1	0	0	1	Complete	
PM1_B4	L-Fucose	carbohydrate	0	1	0	0	2	Complete	
PM1_B7	D,L-a-Glycerol Phosphate	carbohydrate	1	0	0	0	1	Partial	
PM1_B8	D-Xylose	carbohydrate	0	0	1	0	1	Complete	
PM1_C1	D-Glucose-6-Phosphate	carbohydrate	0	0	1	0	1	Partial	
PM1_C4	D-Ribose	carbohydrate	1	0	0	0	2	Complete	
PM1_C6	L-Rhamnose	carbohydrate	0	1	0	0	2	Complete	
PM1_C7	D-Fructose	carbohydrate	1	0	0	0	2	Partial	
PM1_C9	a-D-Glucose	carbohydrate	0	0	1	0	2	Partial	
PM1_D10	Lactulose	carbohydrate	0	0	0	1	2	Absent	
PM1_D11	Sucrose	carbohydrate	0	1	0	0	1	Complete	
PM1_D12	Uridine	carbohydrate	1	0	0	0	1	Complete	
PM1_D8	a-Methyl-D-Galactoside	carbohydrate	0	0	1	0	3	Partial	
PM1_D9	a-D-Lactose	carbohydrate	0	1	0	0	2	Partial	
PM1_E10	Maltotriose	carbohydrate	0	0	1	0	1	Complete	
PM1_E11	2`-Deoxyadenosine	carbohydrate	1	0	0	0	2	Complete	
PM1_E12	Adenosine	carbohydrate	0	0	1	0	2	Complete	
PM1_E3	D-Glucose-1-Phosphate	carbohydrate	0	0	1	0	2	Complete	
PM1_E4	D-Fructose-6-Phosphate	carbohydrate	0	0	1	0	2	Partial	
PM1_E8	b-Methyl-D-Glucoside	carbohydrate	0	0	0	1	3	Absent	
PM1_E9	Adonitol	carbohydrate	0	0	0	1	2	Absent	
PM1_F11	D-Cellobiose	carbohydrate	0	1	0	0	1	Complete	
PM1_F12	Inosine	carbohydrate	0	0	1	0	2	Complete	
PM1_F3	m-Inositol	carbohydrate	0	0	0	1	2	Absent	
PM1_G8	N-Acetyl-D-Mannosamine	carbohydrate	0	0	1	0	2	Complete	
PM1_H5	D-Psicose	carbohydrate	0	0	0	1	2	Absent	
PM1_H6	L-Lyxose	carbohydrate	0	0	0	1	2	Absent	
PM2_B1	N-Acetyl-D-Galactosamine	carbohydrate	0	0	0	1	2	Absent	
PM2_B10	i-Erythritol	carbohydrate	0	0	0	1	2	Absent	
PM2_B11	D-Fucose	carbohydrate	0	0	0	1	2	Absent	
PM2_B12	3-0-b-D-Galactopyranosyl-D-Arab	carbohydrate	0	0	0	1	2	Absent	
PM2_B3	b-D-Allose	carbohydrate	0	0	0	1	1	Absent	
PM2_B4	Amygdalin	carbohydrate	0	0	0	1	1	Absent	
PM2_B5	D-Arabinose	carbohydrate	0	0	0	1	1	Absent	
PM2_B6	D-Arabitol	carbohydrate	0	0	0	1	2	Absent	
PM2_B7	L-Arabitol	carbohydrate	0	0	0	1	2	Absent	
PM2_B8	Arbutin	carbohydrate	0	0	0	1	2	Absent	
PM2_B9	2-Deoxy-D-Ribose	carbohydrate	0	0	0	1	3	Complete	
PM2_C1	Gentiobiose	carbohydrate	0	0	0	1	2	Absent	
PM2_C10	a-Methyl-D-Mannoside	carbohydrate	0	0	0	1	2	Absent	
PM2_C10	a-Methyl-D-Mannoside	carbohydrate	0	0	0	1	5	Absent	
PM2_C11	b-Methyl-D-Xyloside	carbohydrate	0	0	0	1	2	Absent	
PM2_C11	b-Methyl-D-Xyloside	carbohydrate	0	0	0	1	5	Absent	
PM2_C12	Palatinose	carbohydrate	0	0	0	1	3	Absent	
PM2_C12	Palatinose	carbohydrate	0	0	0	1	[illegible]	Absent	
PM2_C2	L-Glucose	carbohydrate	0	0	0	1	2	Absent	

Code	Carbon	Group	Planktonic only	Biofilm only	Planktonic and Biofilm only	Not catabolised	Induce biofilm	Gene	Minimal Media Result
PM2_C3	D-Lactitol	carbohydrate	0	0	0	1	2	Absent	
PM2_C4	D-Lyxose	carbohydrate	0	0	0	1	2	Absent	
PM2_C5	Maltitol	carbohydrate	0	0	0	1	2	Absent	
PM2_C6	a-Methyl-D-Glucoside	carbohydrate	0	0	0	1	1	Absent	
PM2_C7	b-Methyl-D-Galactoside	carbohydrate	0	0	0	1	2	Absent	
PM2_C8	3-Methylglucose	carbohydrate	0	0	0	1	1	Absent	
PM2_D1	D-Raffinose	carbohydrate	1	0	0	0	5	Complete	
PM2_D2	Salicin	carbohydrate	0	0	0	1	5	Absent	
PM2_D3	Sedoheptulosan	carbohydrate	0	0	0	1	4	Absent	
PM2_D4	L-Sorbose	carbohydrate	0	0	0	1	4	Absent	
PM2_D5	Stachyose	carbohydrate	0	0	0	1	3	Complete	
PM2_D6	D-Tagatose	carbohydrate	0	0	0	1	2	Absent	
PM2_D7	Turanose	carbohydrate	0	0	0	1	3	Absent	
PM2_D8	Xylitol	carbohydrate	0	0	0	1	4	Absent	
PM2_D9	L-Xylose	carbohydrate	0	0	0	1	4	Absent	
PM2_F3	Melibionic acid	carbohydrate	1	0	0	0	[illegible]	Partial	
PM1_A4	D-Saccharic acid	carboxylic acid	0	0	0	1	2	Complete	
PM1_A5	Succinic acid	carboxylic acid	1	0	0	0	2	Complete	
PM1_B10	Formic acid	carboxylic acid	0	1	0	0	2	Complete	
PM1_B5	D-Glucuronic acid	carboxylic acid	0	0	1	0	2	Partial	
PM1_B6	D-Gluconic acid	carboxylic acid	1	0	0	0	2	Complete	
PM1_B9	L-Lactic acid	carboxylic acid	0	0	1	0	3	Partial	
PM1_C2	D-Galactonic acid-g-Lactone	carboxylic acid	0	0	0	1	1	Absent	
PM1_C3	D,L-Malic acid	carboxylic acid	0	1	0	0	2	Partial	
PM1_C8	Acetic acid	carboxylic acid	0	1	0	0	2	Complete	
PM1_D3	D-Glucosaminic acid	carboxylic acid	0	0	0	1	2	Absent	
PM1_D6	a-Ketoglutaric acid	carboxylic acid	0	0	0	1	2	Complete	
PM1_D7	a-Ketobutyric acid	carboxylic acid	0	0	1	0	2	Complete	
PM1_E2	m-Tartaric acid	carboxylic acid	0	0	0	1	1	Absent	
PM1_E6	a-Hydroxyglutaric acid-g-Lactone	carboxylic acid	0	0	0	1	2	Absent	
PM1_E7	a-Hydroxybutyric acid	carboxylic acid	1	0	0	0	2	Absent	
PM1_F10	Glyoxylic acid	carboxylic acid	0	0	0	1	1	Complete	
PM1_F2	Citric acid	carboxylic acid	1	0	0	0	2	Complete	
PM1_F5	Fumaric acid	carboxylic acid	0	0	0	1	2	Complete	
PM1_F6	Bromosuccinic acid	carboxylic acid	0	0	0	1	1	Absent	
PM1_F7	Propionic acid	carboxylic acid	0	0	1	0	1	Complete	
PM1_F8	Mucic acid	carboxylic acid	0	0	0	1	1	Absent	
PM1_F9	Glycolic acid	carboxylic acid	0	1	0	0	2	Complete	
PM1_G11	D-Malic acid	carboxylic acid	0	0	0	1	2	Absent	
PM1_G12	L-Malic acid	carboxylic acid	1	0	0	0	2	Absent	
PM1_G2	Tricarballylic acid	carboxylic acid	1	0	0	0	2	Partial	
PM1_G7	Acetoacetic acid	carboxylic acid	0	0	0	1	2	Complete	
PM1_G9	Mono-Methylsuccinate	carboxylic acid	0	1	0	0	1	Partial	
PM1_H10	D-Galacturonic acid	carboxylic acid	0	0	0	1	1	Complete	
PM1_H2	p-Hydroxyphenyl Acetic acid	carboxylic acid	0	0	0	1	2	Absent	
PM1_H3	m-Hydroxyphenyl Acetic acid	carboxylic acid	0	0	0	1	2	Complete	
PM1_H8	Pyruvic acid	carboxylic acid	1	0	0	0	2	Partial	
PM1_H9	L-Galactonic acid-g-Lactone	carboxylic acid	0	0	0	1	1	Absent	
PM2_B2	N-Acetyl-Neuraminic acid	carboxylic acid	1	0	0	0	3	Complete	
PM2_C9	b-Methyl-D-Glucuronic acid	carboxylic acid	0	0	0	1	2	Absent	

Code	Carbon	Group	Planktonic only	Biofilm only	Planktonic and Biofilm only	Not catabolised	Induce biofilm	Gene	Minimal Media Result
PM2_D10	g-Amino-N-Butyric acid	carboxylic acid	0	0	0	1	[illegible]	Complete	
PM2_D11	d-Amino Valeric acid	carboxylic acid	0	0	0	1	[illegible]	Absent	
PM2_D12	Butyric acid	carboxylic acid	0	0	0	1	[illegible]	Absent	
PM2_E1	Capric acid	carboxylic acid	0	0	0	1	[illegible]	Absent	
PM2_E10	b-Hydroxypyruvic acid	carboxylic acid	0	0	0	1	[illegible]	Absent	
PM2_E11	Itaconic acid	carboxylic acid	0	0	0	1	[illegible]	Absent	
PM2_E12	5-Keto-D-Gluconic acid	carboxylic acid	0	0	0	1	[illegible]	Absent	
PM2_E2	Caproic acid	carboxylic acid	0	0	0	1	[illegible]	Absent	
PM2_E3	Citraconic acid	carboxylic acid	0	0	0	1	[illegible]	Absent	
PM2_E4	D,L-Citramalic acid	carboxylic acid	0	0	0	1	[illegible]	Absent	
PM2_E5	Dihydroxyfumaric acid	carboxylic acid	0	0	1	0	[illegible]	Absent	
PM2_E6	2-Hydroxybenzoic acid	carboxylic acid	0	0	0	1	[illegible]	Absent	
PM2_E7	4-Hydroxybenzoic acid	carboxylic acid	0	0	0	1	5	Complete	
PM2_E8	b-Hydroxybutyric acid	carboxylic acid	0	0	0	1	5	Complete	
PM2_E9	g-Hydroxybutyric acid	carboxylic acid	0	0	0	1	4	Absent	
PM2_F10	Succinamic acid	carboxylic acid	0	0	0	1	4	Absent	
PM2_F11	D-Tartaric acid	carboxylic acid	0	0	0	1	5	Absent	
PM2_F12	L-Tartaric acid	carboxylic acid	0	0	0	1	4	Absent	
PM2_F2	Malonic acid	carboxylic acid	0	0	0	1	4	Absent	
PM2_F4	Oxalic acid	carboxylic acid	0	1	0	0	4	Complete	
PM2_F5	Oxalomalic acid	carboxylic acid	0	0	0	1	5	Absent	
PM2_F6	Quinic acid	carboxylic acid	0	0	0	1	4	Absent	
PM2_F7	D-Ribono-1,4-Lactone	carboxylic acid	0	1	0	0	4	Partial	
PM2_F8	Sebacic acid	carboxylic acid	0	0	0	1	4	Absent	
PM2_F9	Sorbic acid	carboxylic acid	0	0	0	1	4	Absent	
PM2_H5	D,L-Carnitine	carboxylic acid	0	0	0	1	[illegible]	Absent	
PM1_G10	Methylpyruvate	ester	1	0	0	0	2	Absent	
PM2_F1	D-Lactic acid Methyl Ester	ester	0	0	0	1	4	Absent	
PM1_C5	Tween 20	fatty acid	0	0	0	1	2	Absent	
PM1_D5	Tween 40	fatty acid	0	1	0	0	2	Absent	
PM1_E5	Tween 80	fatty acid	0	0	0	1	1	Absent	
PM1_A1	Negative Control	negative control	0	0	0	1	1	Absent	
PM2_A1	Negative Control	negative control	0	0	0	1	2	Absent	
PM2_A10	Laminarin	polymer	0	0	0	1	3	Absent	Positive (+)
PM2_A11	Mannan	polymer	0	0	0	1	4	Absent	Positive (+)
PM2_A12	Pectin	polymer	0	0	0	1	5	Absent	Positive (+)
PM2_A2	Chondroitin Sulfate C	polymer	0	0	0	1	4	Absent	Positive (+)
PM2_A3	a-Cyclodextrin	polymer	0	0	0	1	2	Absent	
PM2_A4	b-Cyclodextrin	polymer	0	0	0	1	2	Absent	
PM2_A5	g-Cyclodextrin	polymer	0	0	0	1	2	Absent	
PM2_A6	Dextrin	polymer	1	0	0	0	3	Partial	Positive (+)
PM2_A7	Gelatin	polymer	0	0	0	1	2	Absent	
PM2_A8	Glycogen	polymer	0	0	0	1	3	Complete	Positive (+)
PM2_A9	Inulin	polymer	0	0	0	1	2	Absent	
