# Supplementary material for: Variable Responses to Carbon Utilization between Planktonic and Biofilm Cells of a Human Carrier Strain of Salmonella enterica Serovar Typhi
Source: PLoS One. 2015 May 6;10(5):e0126207. doi: 10.1371/journal.pone.0126207 (PMC4422432; doi:10.1371/journal.pone.0126207)
Supplement: S4 Table — (PDF) [file pone.0126207.s005.pdf]

S4 Table A. Carbon substrates utilized by *Salmonella* Typhi strains in planktonic stage.

No.	PM Code	Carbon Substrates	CR0044	BL191	BL196	S5680	ST33	ST280	STVC1681	STVC3121
1	PM1, A05	Succinic Acid	1	1	1	1	1	0	0	0
2	PM1, B03	Glycerol	0	1	1	1	1	1	1	1
3	PM1, B04	L-Fucose	N/A	N/A	N/A	N/A	N/A	N/A	N/A	N/A
4	PM1, B09	L-Lactic acid	1	1	1	1	1	1	1	1
5	PM1, C06	L-Rhamnose	0	0	0	0	0	0	0	0
6	PM1, C11	D-Melibiose	1	1	1	1	1	1	1	1
7	PM1, F04	D-Threonine	0	0	0	0	0	0	0	0
8	PM1, G03	L-Serine	N/A	N/A	N/A	N/A	N/A	N/A	N/A	N/A
9	PM1, H08	Pyruvic Acid	N/A	N/A	N/A	N/A	N/A	N/A	N/A	N/A
10	PM2, A06	Dextrin	N/A	N/A	N/A	N/A	N/A	N/A	N/A	N/A
11	PM2, A12	Pectin	0	1	1	1	1	1	1	1

[1] Phenotypes were considered as positive growth.

[0] Phenotypes were considered as negative or no growth.

[N/A] Carbon substrate not available.

S4 Table B. Carbon substrates utilized by *Salmonella* Typhi strains in biofilm stage.

No.	PM Code	Carbon Substrates	CR0044	BL191	BL196	S5680	ST33	ST280	STVC1681	STVC3121
1	PM1, A05	Succinic Acid	0	0	0	0	0	0	0	0
2	PM1, B03	Glycerol	1	1	1	1	1	1	1	1
3	PM1, B04	L-Fucose	N/A	N/A	N/A	N/A	N/A	N/A	N/A	N/A
4	PM1, B09	L-Lactic acid	1	1	1	1	1	1	1	1
5	PM1, C06	L-Rhamnose	1	0	0	0	0	0	0	0
6	PM1, C11	D-Melibiose	1	1	1	1	1	1	1	1
7	PM1, F04	D-Threonine	1	0	0	0	0	0	0	0
8	PM1, G03	L-Serine	N/A	N/A	N/A	N/A	N/A	N/A	N/A	N/A
9	PM1, H08	Pyruvic Acid	N/A	N/A	N/A	N/A	N/A	N/A	N/A	N/A
10	PM2, A06	Dextrin	N/A	N/A	N/A	N/A	N/A	N/A	N/A	N/A
11	PM2, A12	Pectin	0	0	0	0	0	0	0	0

[1] Phenotypes were considered as positive growth.

[0] Phenotypes were considered as negative or no growth.

[N/A] Carbon substrate not available.

S4 Table C. Carbon substrates utilized by *Salmonella* Typhi strains during transition from planktonic to biofilm stage.

No.	PM Code	Carbon Substrates	CR0044	BL191	BL196	S5680	ST33	ST280	STVC1681	STVC3121
1	PM1, B03	Glycerol	0	0	0	0	0	0	0	0
2	PM2, A06	Dextrin	N/A	N/A	N/A	N/A	N/A	N/A	N/A	N/A
3	PM2, A12	Pectin	1	0	0	0	0	0	0	0

[1] Phenotypes were considered as positive growth.

[0] Phenotypes were considered as negative or no growth.

[N/A] Carbon substrate not available.
